# Borylcyclopropanes: Synthetic Strategies and Applications

**DOI:** 10.1002/anie.8294925

**Published:** 2026-07-23

**Authors:** Aiza A. Butt, Joseph M. Ready

**Affiliations:** ^1^ Department of Biochemistry Division of Chemistry UT Southwestern Medical Center Dallas Texas USA

**Keywords:** borylcyclopropane synthesis, C─B bond functionalization, cyclopropanation, organoboron chemistry, stereoselective synthesis

## Abstract

The carbon–boron bond is among the most versatile functional groups in organic synthesis, and its installation within the strained cyclopropane framework generates borylcyclopropanes with a rich reactivity profile. The presence of a boron moiety on the cyclopropane ring unlocks a broad spectrum of transformations inaccessible to unfunctionalized cyclopropanes, including Suzuki–Miyaura cross‐coupling, oxidation, amination, Matteson homologation, and stereospecific ring‐opening via 1,2‐metalate rearrangement to enantioenriched acyclic organoboron arrays. Meanwhile, the distinctive reactivity of boron‐stabilized intermediates such as α‐boryl radicals, α‐boryl carbanions, and α‐boryl cations enables cyclopropanation strategies with no classical counterpart. This review provides an exhaustive survey of borylcyclopropane chemistry, organized by mechanistic class: metal–carbene cyclopropanations under palladium, copper, rhodium, and enzyme catalysis; non‐diazo carbene and carbenoid approaches; transition‐metal‐catalyzed and metal‐free intramolecular nucleophilic cyclizations; photocatalytic radical pathways; iridium‐ and palladium‐catalyzed C─H borylation; hydroboration of cyclopropenes and alkylidenecyclopropanes; and miscellaneous strategies including bicyclo[1.1.0]butane (BCB) ring‐opening and 1,2‐metalate rearrangements. Applications in medicinal chemistry, natural product synthesis, and the preparation of stereodefined acyclic building blocks are also discussed.

## Graphical abstract

Borylcyclopropanes (cyclopropanes bearing a boron substituent) sit at the intersection of two privileged frameworks in synthesis. This review provides the first exhaustive survey of their chemistry, spanning metal–carbene, nucleophilic cyclization, radical, hydroboration, and C─H activation routes, and documenting applications in medicinal chemistry, natural product synthesis, and stereospecific ring‐opening and strain‐release transformations.

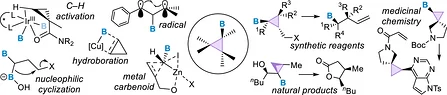

## 1 Introduction

**SCHEME 1:**
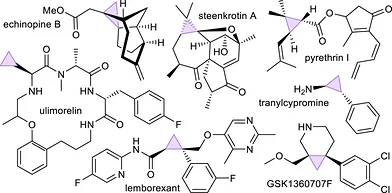
Examples of cyclopropane‐containing natural products and drugs.

Cyclopropanes are among the most synthetically valuable carbocyclic frameworks in modern organic chemistry. The rigid, strained architecture, and unique electronic properties make cyclopropane a privileged scaffold in natural products, pharmaceutical agents, and agrochemicals, such as echinopine B, steenkrotin A, and pyrethrin I, and approved drugs such as tranylcypromine, ulimorelin, and lemborexant (Scheme 1) [1, 2, 3, 4]. Beyond their biological relevance, cyclopropyl units serve as conformationally restricted building blocks in drug discovery, where they modulate metabolic stability, binding affinity, and rigidity during structure–activity relationship (SAR) campaigns [4]. Tri‐ and disubstituted variants appear broadly in bioactive natural products, while certain cyclopropane‐containing peptide mimetics act as amino acid isosteres in β‐turn and hairpin structures [5, 6].

Organoboron compounds occupy an equally central role in organic synthesis. Alkylboronic esters are exceptionally versatile intermediates whose carbon–boron bond can be functionalized through an expansive set of transformations, including Suzuki–Miyaura cross‐coupling, hydroboration, allylboration, 1,2‐metalate rearrangements, oxidation, amination, halogenation, protodeboronation, and Zweifel olefination [7, 8, 9]. Boron can stabilize adjacent radicals [10] and anions [11, 12], and the C─B bond can be homolytically cleaved to generate alkyl radicals [13, 14]. The clinical approval of boron‐containing drugs such as bortezomib, tavaborole, and crisaborole further underscores the therapeutic relevance of this compound class [15].

At the intersection of these two domains, borylcyclopropanes have emerged as a uniquely powerful family of building blocks. The value of borylcyclopropanes stems from their versatile C─B bonds, which unlock reactivity manifolds inaccessible to simple cyclopropanes. Boron functionality enables both their formation and selective transformations into other products through boron's tunable polarity and coordination aptitude. For instance, cyclopropylboronic esters can engage in transition‐metal‐catalyzed cross‐coupling with nucleophiles, facilitating late‐stage installation of the cyclopropyl motif onto complex molecules. More distinctive still are transformations that exploit the ambiphilic character of *gem*‐diborylalkanes: these species access both carbanion‐like and carbocation‐like intermediates, enabling stereospecific deborylative alkylations through boronate complexes [12, 16, 17, 18], [2 + 1] cyclization with α‐halodiborylmethyl carbanion (also known as “boron ylides”) [19, 20, 21], borata alkene‐induced ring‐opening [22], as well as [1, 3]‐hydride shifts via α‐boryl cations [23]. While access to boronic esters has historically relied on the electrophilic nature of boron (through hydroboration or addition of organometallics to borate esters) nucleophilic intermediates such as α‐boryl carbanions (also referred to as boron alkylidenes or borata alkenes) enable fundamentally opposite reactivity patterns [24, 25, 26]. Copper‐boryl complexes represent another such class: the high‐energy Cu─B σ* orbital favors interaction of the filled Cu─B σ‐bond with the LUMO of the substrate, imparting strong nucleophilicity inaccessible with other transition metals and enabling C─B bond formations previously unattainable [27, 28]. α‐ or β‐Boryl radical intermediates have similarly been exploited in cyclopropanation strategies [29, 30, 31, 32, 33, 34]. α‐Boryl radicals benefit from stabilization through electron delocalization into the adjacent boron p‐orbital [10, 35, 36, 37], while β‐boryl radicals are stabilized through hyperconjugation [38, 39, 40, 41]. Boron stabilized carbenes and carbenoids constitute yet another distinctive class: α‐boryl carbenes, accessible through chromium [42, 43] and copper [44] catalysis, and α‐boryl carbenoids like boromethylzinc carbenoids [45] represent reactive intermediates whose behavior is fundamentally governed by the electronic influence of the adjacent boron atom. Collectively, this reactivity profile expands the synthetic toolkit available for both the construction and elaboration of borylcyclopropanes, enabling selective transformations that complement or surpass what classical cyclopropane chemistry alone can achieve.

Two prior reviews have tangentially addressed aspects of this chemistry: Martín‐Heras, Parra, and Tortosa's 2018 account [46] treated cyclopropylboronic esters as one subclass within a broader discussion of strained organoboron compounds, while Kang, Lv, and Shi's recent *Chemical Society Reviews* contribution [47] examined cyclopropane borylation in the context of C─H and C─C bond activation. Neither manuscript provides a comprehensive treatment of the field. The present review fills this gap, offering an in‐depth survey of borylcyclopropane chemistry, covering the extensive range of synthetic routes reported to date. A related review by Das [48] appeared in *Advanced Synthesis & Catalysis* in 2026, while this manuscript was under review, and likewise highlights selected aspects of borylcyclopropane chemistry.

**SCHEME 2:**
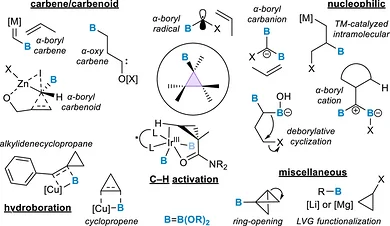
Overview of borylcyclopropanation methods.

Representative synthetic routes to borylcyclopropanes are illustrated in Scheme 2. We begin with carbene and carbenoid approaches—encompassing diazo‐mediated metal‐carbene cyclopropanations with Pd, Cu, Rh, and enzyme catalysts, as well as non‐diazo variants including Simmons–Smith and transition‐metal carbene‐mediated routes—before turning to intramolecular nucleophilic ring closure strategies, C─H activation, and hydroboration of cyclopropenes and alkylidenecyclopropanes. Miscellaneous methods, including bicyclo[1.1.0]butane ring opening, leaving group substitution, and 1,2‐metalate rearrangements, are also discussed. The review concludes with an account of the synthetic applications of borylcyclopropanes, spanning medicinal chemistry, natural product synthesis, and their use as precursors to enantioenriched acyclic structures, providing a unified and definitive reference for this rapidly growing field.

## 2 Carbene and Carbenoid Routes to Borylcyclopropanes

### 2.1 Diazo‐Mediated Metal–Carbene Borylcyclopropanations

Carbene and carbenoid intermediates are central to the most classical and widely employed strategies for cyclopropane synthesis, and their application to borylcyclopropane formation has been extensively developed. Diazo compounds have been most frequently explored as carbene precursors, with various transition metal catalysts and even enzyme‐catalyzed routes having been developed, each dictating the reactivity, selectivity, and substrate scope of the transformation.

#### 2.1.1 Palladium‐Catalyzed Reactions

**SCHEME 3:**
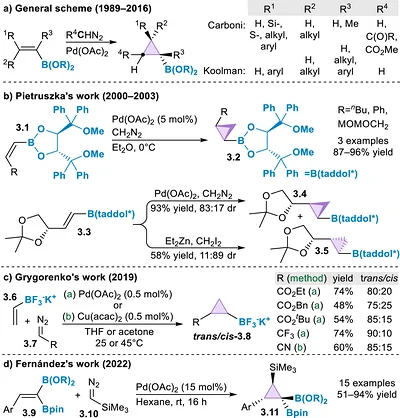
Palladium‐catalyzed transformations via diazo‐derived carbenes.

The first Pd‐catalyzed borylcyclopropanation was introduced by Fontani and coworkers in 1989, combining vinylboronic esters with diazomethane or methyl diazoacetate to synthesize borylcyclopropanes (Scheme 3a) [49]. The methylene transfer proceeded with conservation of the olefin stereochemistry, and mono‐ and disubstituted alkenes gave good yields, whereas trisubstituted substrates were incompatible. The method was not limited to methylene transfer, as the methoxycarbonylcarbene generated from methyl diazoacetate delivered the corresponding ester‐substituted borylcyclopropanes. Building on this carbene approach, the same group expanded the scope of the reaction in 1991 to acyl carbenes generated from 2‐diazo‐1‐phenyl‐1‐ethanone, delivering *cis/trans* mixtures of disubstituted borylcyclopropanes [50]. Compared to the Simmons–Smith alternative detailed in the same study, this Pd‐catalyzed method proved more sensitive to steric hindrance.

A significant advance in stereochemical control came in 2000, when Pietruszka and coworkers introduced stable, enantiomerically pure cyclopropylboronic esters (**3.1**) derived from the TADDOL‐based chiral auxiliary, (2*R*,3*R*)‐1,4‐dimethoxy‐1,1,4,4‐tetraphenyl‐2,3‐butanediol, overcoming the lability of prior literature and enabling easier isolation, purification, and storage (Scheme 3b) [51]. While earlier protocols reliably synthesized *trans*‐1,2‐disubstituted cyclopropanes, this work established the first synthesis of *cis*‐configured borylcyclopropanes (**3.2**). In a follow‐up study in the same year, the group examined the effect of introducing an additional stereogenic center as a substituent on the borylcyclopropane bound to the TADDOL‑based chiral auxiliary [52]. In these systems, cyclopropanation of alkenylboronic ester **3.3** with diazomethane and palladium(II) acetate was predominantly auxiliary‐controlled, giving **3.4** as the major diastereomer, whereas the Simmons–Smith protocol operated mainly under substrate control, favoring the alternative diastereomer **3.5**.

Seeking alternatives to the previously studied boronic esters, Pietruszka et al. identified commercially available benzpinacol (1,1,2,2‐tetraphenyl‐1,2‐ethanediol) in 2002 as a diol that forms highly stable boronic esters that showed negligible hydrolysis under the conditions examined [53]. They outperformed both 1,3,2‐dioxaborinanes/‐dioxaborolanes and conventional pinacol boronic esters, providing substrate‐controlled borylcyclopropanation. The next year, the same group developed “Garner” aldehyde‐derived borylcyclopropanes, from which they derived cyclopropane‐containing amino acids (see Section 7.1, Scheme 28a) [54]. In 2002, Marko ´et al. expanded the Pd(OAc)_2_/CH_2_N_2_‐catalyzed cyclopropanation to functionalized dienes, utilizing various substituted dienylboronic esters to synthesize 1‐boronato‐2‐vinylcyclopropanes in moderate‐to‐good yields [55].

Addressing the hazards associated with diazomethane handling, Koolman et al. developed an automated diazomethane flow reactor based on a permeable tube‐in‐tube concept in 2016 [56]. This system makes Pd‐catalyzed borylcyclopropanations more accessible, enabling repeated small‐scale and large‐scale reactions without managing large quantities of hazardous diazomethane solutions. The technology was used to prepare a library of cyclopropyl boronic esters to support various medicinal chemistry programs.

Since the cyclopropylboronic esters of chiral diols that Pietruszka's group developed were reluctant to engage in cross‐coupling reactions, Deng and coworkers developed a route to stereodefined cyclopropyl trifluoroborates and demonstrated their cross‐coupling reactions with aryl bromides [57]. These cyclopropyl trifluoroborates **3.8** could also be accessed from vinyltrifluoroborate **3.6**, as demonstrated in 2019 by Grygorenko and coworkers through an expanded Pd‐catalyzed cyclopropanation platform (Scheme 3c) [58]. This study broadened the range of diazoalkanes suitable for Pd‐catalyzed cyclopropanation, enabling a practical multigram‐scale synthesis of functionalized 1,2‐disubstituted cyclopropyl trifluoroborates **3.8**, and represented the first approach to these molecules that did not proceed via their corresponding boronic esters. Each diazoalkane (**3.7**), including ethyl diazoacetate, benzyl diazoacetate, and others, required individual optimization. Pd(OAc)_2_ proved optimal for most substrates, while diazoacetonitrile demanded Cu(acac)_2_ instead. These conditions delivered moderate to good *trans* diastereoselectivity, allowing efficient isolation of the major isomer by recrystallization. In 2022 [59], Fernández and coworkers described a palladium‐catalyzed silylcyclopropanation of 2‐aryl 1,1‐diborylalkenes (**3.9**) with (trimethylsilyl)diazomethane (**3.10**), inspired by the Fontani group's work in 1989 [49] (Scheme 3d). The 1,1‐bis(pinacolboryl)alkenes were prepared via two routes: (i) condensation of tris(boryl)methane with aldehydes involving B─O elimination, or (ii) copper‐catalyzed dehydrogenative borylation/hydroboration of alkynes with pinacolborane. Some of these diborylalkenes underwent further stereoselective transborylation. Using (trimethylsilyl)diazomethane with Pd(OAc)_2_ for 16 h generated *gem*‐diborylcyclopropanes with complete *anti* diastereoselectivity between the trimethylsilyl and aryl groups, potentially via migratory insertion of Pd═CH‐TMS into the trisubstituted alkene. The scope of these polyfunctionalized B,B,Si‐cyclopropanes (**3.11**) included functionalized phenyl and naphthyl rings, as well as varied boryl groups such as (+)‐pinanediolboryl (Bpai), which afforded ≥90:10 dr.

#### 2.1.2 Copper‐Catalyzed Reactions

**SCHEME 4:**
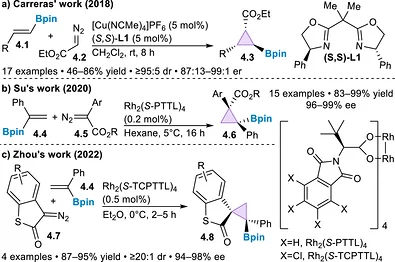
Copper‐ and rhodium‐catalyzed transformations via diazo‐derived carbenes.

Copper catalysis was first applied to the cyclopropanation of alkenyl boronic esters by Carreras et al. in 2018, who achieved highly enantio‐ and diastereoselective synthesis of 1‐boryl‐2,3‐disubstituted cyclopropanes (**4.3**) from alkenylboronic esters **4.1** and ethyl diazoacetate **4.2** (Scheme 4a) [60]. The concerted carbene transfer preserved the original *E/Z* geometry of the alkene, so the relative arrangement of the aryl and boryl groups was retained in the resulting borylcyclopropanes. Single‐crystal x‐ray diffraction analysis established the relative and absolute stereochemistry of the products. Across the scope, *ortho‐*, *meta‐*, and *para‐*substituted arenes, thiophene, and alkyl chains were all well tolerated, providing good yields with consistently excellent diastereoselectivity (94:6 to 97:3 dr) and enantioselectivity (94:6 to 99:1 er) induced by chiral bisoxazoline ligands **L1**; only *Z‐*alkenylboronic esters showed a drop in er below 90:10. In addition, the authors developed a straightforward alkyne hydroboration to access *E‐*alkenylboronic esters, and demonstrated that a one pot sequential hydroboration/borylcyclopropanation could be performed in good overall yields (61%–79%). In 2021, the same group extended this methodology to trifluorodiazoethane, enabling the synthesis of 2‐substituted‐3‐(trifluoromethyl)cyclopropylboronic esters with high levels of stereocontrol [61].

In 2022, Mali and coworkers attempted to adapt the Carreras methodology to access tetrasubstituted bisborylcyclopropanes from a pinacol 1,2‐diboronic ester and ethyl diazoacetate, but obtained only moderate yield (42%) and low enantioselectivity (34% ee) [62]. Consequently, the authors redirected their efforts toward their Simmons–Smith protocol to access closely related target structures instead (see Section 2.2.1).

#### 2.1.3 Rhodium‐Catalyzed Reactions

Rhodium catalysis offered a complementary approach, with Sun and coworkers reporting in 2020 a diastereo‐ and enantioselective cyclopropanation of α‐boryl styrenes **4.4** with α‐diazoarylacetates **4.5** (Scheme 4b) [63]. They highlighted improvements over the 2018 Carreras report, employing far less catalyst (0.2 mol% Rh vs. 5 mol% Cu) and avoiding restriction to a single diazo compound. Optimal enantioselectivity, diastereoselectivity, and yields of **4.6** were achieved with Rh_2_(*S*‐PTTL)_4_. The reaction tolerated diverse functional groups on the aryl groups of both reaction partners, including a naphthyl ring from the aryldiazoacetate. In 2022, Zhou and coworkers presented a similar highly stereoselective Rh‐catalyzed olefin cyclopropanation of diazothiooxindoles (**4.7**) to access optically active spirocyclopropylthiooxindoles (**4.8**) via a broad range of α‐functionalized styrenes (**4.4**), including of α‐boryl styrenes (Scheme 4c) [64].

#### 2.1.4 Enzyme‐Catalyzed Reactions

**SCHEME 5:**
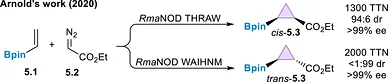
Enzyme‐catalyzed transformations via diazo‐derived carbenes.

The most striking advance in this area came from enzymatic catalysis: Arnold and coworkers pioneered in 2020 the biocatalytic borylcyclopropanation through engineering of *Rhodothermus marinus* nitric oxide dioxygenase (*Rma*NOD) variants that catalyze highly diastereo‐ and enantioselective carbene transfer from ethyl diazoacetate (**5.2**) to vinylboronic acid pinacol ester (**5.1**), accessing either *cis*‐ or *trans*‐borylcyclopropane diastereomers (Scheme 5) [65]. Building on their prior discovery that heme proteins enable cyclopropanation via carbene transfer [66], the authors screened numerous heme variants (cytochromes P411, cytochromes c, and globins) but identified only *Rma*NOD‐Q52A [67] as active, albeit with low efficiency. Iterative single‐site saturation mutagenesis and recombination of the distal heme pocket then yielded *Rma*NOD‐Y32T‐Y39H‐L48R‐Q52A‐R79W (*Rma*NOD‐THRAW; 1300 TTN, 94:6 dr, >99% ee) for *cis*‐**5.3**, and *Rma*NOD‐L20W‐Q52A‐L56I‐L60H‐L101N‐I105M (*Rma*NOD‐WAIHNM; 2000 TTN, <1:99 dr, >99% ee) for *trans*‐**5.3**. To improve access to the catalyst formulation, the authors found that lyophilized *Rma*NOD‐THRAW and *Rma*NOD‐WAIHNM in whole *E. coli* cells resuspended in aqueous buffer supported preparative‐scale reactions. Furthermore, efficient isolation of the borylcyclopropane, hindered by chromatography‐incompatible impurities, was achieved by derivatizing the crude extract to the corresponding trifluoroborate salt for straightforward separation.

### 2.2 Non‐Diazo Carbene and Carbenoid Borylcyclopropanations

#### 2.2.1 Simmons–Smith Zinc Carbenoid Mediated Reactions

While diazo compounds have dominated carbene‐based borylcyclopropanation, non‐diazo routes offer distinct practical advantages, most notably avoiding the toxicity and explosion hazards associated with diazo reagents. The Simmons–Smith reaction is the most classical among these, proceeding through a zinc carbenoid intermediate to deliver *syn*‐stereospecific methylene transfer under mild, bench‐stable conditions.

**SCHEME 6:**
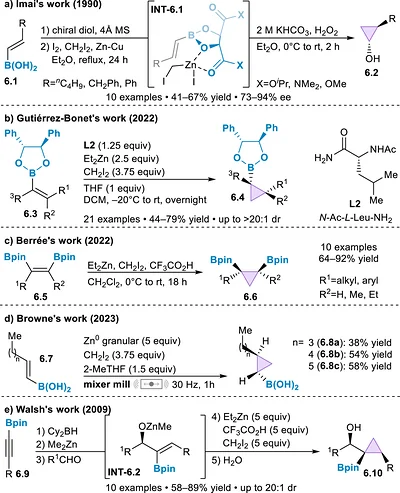
Simmons–Smith zinc carbenoid mediated reactions from borylated alkenes and alkynes.

In 1990, Imai et al. introduced the first asymmetric borylcyclopropanation of 1‐alkenylboronic esters via a diastereofacially selective Simmons–Smith reaction (Scheme 6a) [68]. The alkenylboronic acids (**6.1**) were derivatized with chiral diols (+)‐diisopropyl tartrate (DIPT), (+)‐*N,N,N’,N’*‐tetramethyltartaramide (TMTA), and (+)‐dimethyl tartrate (DMT) to form enantiopure 1‐alkenylboronic esters, which underwent cyclopropanation under Simmons–Smith conditions via intermediate **INT‐6.1** to form optically active cyclopropylboronic esters, followed by direct oxidation to deliver optically active cyclopropanols (**6.2**) in moderate yields (41%–67%) and good ee's (73%–94%). The authors rationalized the diastereofacial selectivity through chelation of the Simmons–Smith reagent by the substrate prior to methylene transfer.

Several papers from the 1990s–2000s [50, 51, 52, 53, 54] mentioned in the diazomethane–Pd(OAc)_2_ section of this review also examined the Simmons–Smith reaction, observing similar reactivity profiles. A key distinction between the two methods was the greater sensitivity of palladium catalysis to steric hindrance, with trisubstituted vinylboronic esters reacting smoothly under Simmons–Smith conditions but proving incompatible with the diazo/palladium system [50]. Additionally, some examples showed opposing stereochemical control modes: in Pietruszka's studies, stereoselectivity in Simmons–Smith cyclopropanation was dictated by an allylic stereocenter while palladium catalysis was governed by the chiral auxiliary on boron (see Scheme 3b above) [52].

The methodologies developed by the Imai and Pietruszka groups were both limited to using 1‐alkenylboronic esters, and thus, synthesizing 1,2‐disubstituted borylcyclopropanes. In 2022, Gutiérrez‐Bonet et al. expanded these studies to tertiary and secondary cyclopropyl boronic esters (**6.4**) by using internal and terminal enantioenriched alkenyl boronic esters (**6.3**), including cycloalkenyl boronic esters, under classic Simmons−Smith conditions (Scheme 6b) [69]. The alkenylboronic acids were condensed with (*S,S*)‐hydrobenzoin to form their boronic ester counterparts (**6.3**); among various Lewis basic ligands tested, *N*‐Ac‐*L*‐Leu‐NH_2_ (**L2**) provided the highest borylcyclopropane diastereoselectivity (from 1:1.4 to 6.2:1 dr), favoring the *syn* diastereomer.

Building on these methodologies by modifying Simmons–Smith conditions to enable cyclopropanation of alkenyl‐1,2‐bis(boronic esters) **6.5**, Mali and coworkers in 2022 stereoselectively delivered doubly borylated cyclopropanes **6.6** (Scheme 6c) [62]. Treatment with diiodomethane and Et_2_Zn in the presence of trifluoroacetic acid at room temperature in CH_2_Cl_2_ furnished 1,2‐substituted cyclopropyl bisboronic esters (**6.6**) in good yields (64%–92%). NOESY analysis confirmed retention of configuration, with broad tolerance of alkyl‐substituted tri‐ and tetrasubstituted alkenes, including one phenyl‐substituted example.

More recently, Pontini, Leitch, and Browne demonstrated that mechanochemical activation of zinc(0) via ball‐milling could enable a solvent‐free Simmons–Smith cyclopropanation under air without recourse to air/moisture‐sensitive setups (Scheme 6d) [70]. Within the broad substrate scope, vinyl boronic acids (**6.7**) were shown to be compatible with the reaction conditions, delivering cyclopropyl boronic acid products (**6.8a–c**) in 38%–58% yield, providing handles for downstream cross‐coupling chemistry. Although modest in yield, this represents a practical, sustainable entry to borylcyclopropanes under uniquely mild and operationally simple conditions, and the protocol was demonstrated to be scalable to gram scale. Shifting to alkynes, earlier in 2009, Walsh and coworkers reported a one‐pot synthesis of cyclopropyl alcohol boronic esters (**6.10**) from a 1‐alkenyl‐1,1‐borozinc heterobimetallic intermediate in good isolated yields and excellent diastereoselectivities (Scheme 6e) [71]. The sequence began with hydroboration of Bpin‐substituted alkynes (**6.9**) using dicyclohexylborane, followed by rapid, chemoselective metalation with dialkylzinc to generate the boron/zinc 1,1‐heterobimetallic complex. These mild, functional‐group‐tolerant reagents then underwent carbonyl addition via the more reactive Zn─C bond to afford a Bpin‐substituted allylic alkoxide intermediate **INT‐6.2**, which in turn underwent an in situ, alkoxide‐directed cyclopropanation to furnish the desired cyclopropyl boronic esters in good to excellent yields. The authors further showcased this methodology in the synthesis of quercus lactones A and B (see Section 7.2, Scheme 29b).

**SCHEME 7:**
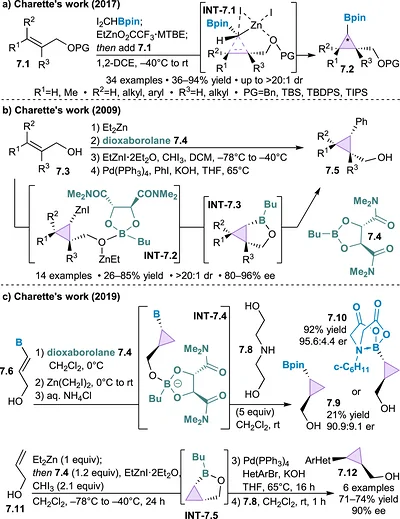
Simmons–Smith zinc carbenoid mediated reactions from alkenes.

In 2017, Benoit and Charette reported a borylcyclopropanation of (*E*)‐ and (*Z*)‐allylic ethers and styrene derivatives (**7.1**) using a Simmons–Smith reaction with a novel boromethylzinc carbenoid (Scheme 7a) [45]. The diiodomethylpinacol boronic ester precursor to this α‐boryl carbenoid (**INT‐7.1**) was prepared on multigram scale in three steps from dichloromethane. Extensive optimization of the zinc reagent identified ethylzinc trifluoroacetate as optimal, with methyl *tert*‐butyl ether (MTBE) as a superior complexing additive. The reaction tolerated a broad range of alkenes: electron‐rich cinnamyl ethers furnished 1,2,3‐trisubstituted borylcyclopropanes (**7.2**) in excellent yields and diastereoselectivities, even in the presence of sensitive functional groups such as acetates, alcohols, and azides, whereas electron‐poor analogues, secondary benzyl ethers, and styrenes were significantly less reactive. Alkyl‐substituted and trisubstituted olefins were also well tolerated, although high diastereocontrol required a 1,2‐disubstituted alkene. Compatible protecting groups included benzyl and a variety of silyl ethers. The observed diastereoselectivities are consistent with minimizing steric interactions between the pinacolborane substituent and R^1^ or R^2^, favoring either a *cis* or *trans* relationship between Bpin and the allylic ether oxygen. The proposed stereochemical model is also consistent with the greater diastereocontrol observed with benzyl ethers relative to styrenes, and with the requirement of a 1,2‐disubstituted alkene for diastereocontrol.

Earlier in 2009, Zimmer and Charette developed an asymmetric zinco‐cyclopropanation of allylic alcohols **7.3** via a cyclopropyl borinate species (Scheme 7b) [72]. The reaction proceeded via deprotonation of the allylic alcohol by Et_2_Zn, followed by the reaction of the zinc alkoxide with chiral dioxaborolane‐based reagent (**7.4**) to generate a tetracoordinated boronate species. This intermediate reacted with *gem*‐dizinc carbenoid to generate cyclopropylzinc (**INT‐7.2**), which produced cyclopropyl borinate **INT‐7.3** by boron–zinc exchange. Instead of isolating the borinate species, the authors proceeded directly to Suzuki–Miyaura cross‐coupling reaction to yield 1,2,3‐trisubstituted cyclopropanes (**7.5**) in good yields with good‐to‐excellent enantioselectivities.

Extending the usefulness of chiral reagent **7.4** to the cyclopropanation of alkenyl boronic esters, the same group reported an enantioselective synthesis of *cis*‐ and *trans*‐borocyclopropylmethanols in 2019 (Scheme 7c) [73]. Using either CIDA‐ or Bpin‐substituted allylic alcohol (**7.6**), cyclopropanation with Zn(CH_2_I)_2_ in the presence of tartaramide‐based chiral dioxaborolane **7.4** afforded the corresponding boronate complex **INT‐7.4**. Subsequent treatment with diethanolamine (**7.8**) as a non‐oxidative decomplexation reagent provided the *trans* Bpin‐ and CIDA‐based borocyclopropylmethanols (**7.9** and **7.10**) in 21% and 92% yield, respectively, both with high enantioselectivity. Because *cis* CIDA‐substituted allylic alcohols could not be prepared due to their instability, the authors instead used an in‐situ approach examined in their 2009 report [72] with allyl alcohol **7.11** that proceeds via cyclic borinate **INT‐7.5**. This intermediate was subjected to Suzuki cross‐coupling with *N*‐heterocycles to give *cis*‐*N*‐heterocycle‐substituted cyclopropanes **7.12** in good yields (71%–75%) and excellent enantioselectivities. Overall, this study extended the author's 2009 report by providing access to the *trans*‐substituted boryl cyclopropanes and expanding the scope of the Suzuki–Miyaura coupling to accommodate heteroarenes.

Despite their efficiency and elegance, some of these methods require pyrophoric or toxic reagents such as diethylzinc, which hinders large‐scale applications.

#### 2.2.2 Chromium Carbene Mediated Reactions

**SCHEME 8:**
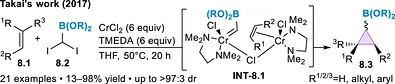
Chromium and nickel carbene mediated reactions.

Extending their earlier iodo‐ and silylcyclopropanation of terminal olefins to the boron setting, Takai et al. reported a chromium‐mediated α‐boryl carbene cyclopropanation in 2007 [42]. Preliminary studies showed that allyl benzyl ether and 1‐dodecene underwent cyclopropanation with dichloromethylboronic ester (**8.2**) in the presence of CrCl_2_, LiI, and TMEDA to furnish the corresponding borylcyclopropanes in 75% yield, albeit with low *E/Z* stereoselectivity (1.1:1 and 1.7:1 dr, respectively). A decade later, the same group achieved a significant stereochemical improvement through a chromium‐promoted α‐boryl carbene cyclopropanation proceeding via a *gem*‐dichromiomethane intermediate (Cr─CHR─Cr), which, unlike classical Simmons–Smith chemistry, enabled efficient cyclopropanation of unactivated alkenes lacking heteroatom directing groups (Scheme 8) [43]. Earlier that year, Charette and coworkers had shown that Simmons–Smith‐type borylcyclopropanation is largely restricted to allylic ethers and aryl‐substituted olefins [45], and the Takai group specifically sought to overcome this limitation by targeting unactivated alkenes (**8.1**). Using diiodomethylboronic acid pinacol ester (I_2_CHBpin, **8.2**) with CrCl_2_, they identified TMEDA as the optimal ligand, since other nitrogen‐ and phosphine‐based ligands diminished the yield. Besides pinacol, neopentylglycol and pinanediol boronic esters were also compatible. Cyclic, linear, and monosubstituted olefins gave good yields, whereas *E*‐alkenes performed poorly and trisubstituted alkenes were not tolerated. In contrast to Simmons–Smith conditions, which typically favor 1,1‐disubstituted and trisubstituted alkenes over *cis*‐disubstituted ones, this chromium system was chemoselective for the *cis*‐disubstituted double bond in substrates bearing multiple alkenes. The proposed mechanism for (*Z*)‐disubstituted alkenes involves reduction of the diiodomethylboronic ester by [(TMEDA)CrCl_2_]_2_ to a *gem*‐dichromiomethylboronate, formation of a chromocarbene intermediate **INT‐8.1**, and [2+2] cycloaddition with the alkene to give a chromocyclobutane, followed by reductive elimination of chromium to furnish the borylcyclopropane **8.3**.

#### 2.2.3 Nickel Carbene Mediated Reactions

**SCHEME 9:**
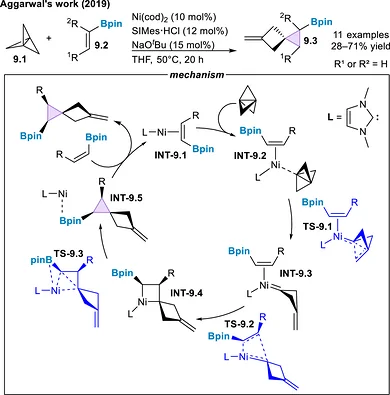
Nickel carbene mediated reactions.

An unusual carbene source was reported in 2019, when Yu and coworkers disclosed a nickel‐catalyzed cyclopropanation that employs [1.1.1]propellane (**9.1**) as a carbene precursor to furnish methylenespiro[2.3]hexane products, including borylated variants (Scheme 9) [74]. The authors found that Ni(cod)_2_ selectively promoted cyclopropanation, whereas the corresponding Ir(I), Pd(0), and Cu(I) systems favored isomerization and oligomerization instead. The reaction scope initially proved narrow, being effective only for aryl‐ and heteroaryl‐substituted olefins as well as conjugated 1,3‐dienes and 1,3‐enynes. The method was not effective with alkyl‐substituted alkenes or with α‐ or β‐substituted styrenes. To address this limitation, the authors evaluated alkenylboronic esters (**9.2**): vinylboronic acid pinacol esters, as well as α‐ and β‐alkyl‐substituted alkenylboronic esters, underwent productive reaction to give borylated methylenespiro[2.3]hexanes (**9.3**) in moderate to good yields and modest *trans/cis* diastereoselectivity, whereas trisubstituted alkenes remained unreactive. Mechanistic experiments concluded that the reaction proceeded via a two‐electron pathway involving reaction of the alkene substrate with a nickel carbene intermediate. DFT studies were used to model the catalytic cycle, in which a simplified dimethyl NHC was used as a representative ligand. The 14‐electron intermediate **INT‐9.1** coordinates to [1.1.1]propellane to afford the thermodynamically favored Ni(0) adduct **INT‐9.2**. This then undergoes a concerted ring opening in which two C─C bonds are cleaved to furnish carbene intermediate **INT‐9.3** via **TS‐9.1**. Subsequent cyclopropanation proceeds in a step‐wise manner through formation of a metallocyclobutane intermediate **INT‐9.4** via **TS‐9.2**, followed by reductive elimination through **TS‐9.3** to give the Ni(0)–product complex **INT‐9.5**. Displacement of the product by a new alkenyl‐Bpin substrate regenerates **INT‐9.1**. The Bpin moiety dramatically expanded the scope of this method by enabling reactivity with 1,2‐ and 1,1‐disubstituted olefins and alkyl‐substituted olefins, all of which were unreactive in non‐boryl substrates.

#### 2.2.4 Copper Carbene Mediated Reactions

**SCHEME 10:**
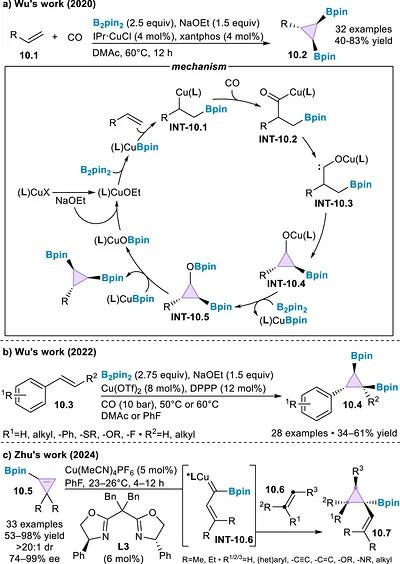
Copper carbene mediated reactions.

The Wu group reported an unusual Cu‐catalyzed cyclopropanation in 2020 that involved carbonylation of terminal olefins (Scheme 10a) [75]. Overall, the method converts unactivated aliphatic alkenes into 1,2‐diborylated cyclopropanes. Various aliphatic terminal alkenes (**10.1**), including those with an additional olefin, as well as natural product derivatives afforded trisubstituted vicinal diborylcyclopropanes (**10.2**) in moderate‐to‐good yield, whereas styrene proved unsuitable and instead predominantly underwent hydroboration. A mechanistic control experiment generated the β boryl alkylcopper(I) complex (**INT‐10.1**) in situ by alkene insertion into the (L)Cu‐Bpin species; upon subsequent addition of B_2_pin_2_ and CO gas, the desired product was obtained, thereby confirming this complex as a key intermediate on the pathway to the carbonylative borylcyclopropanation. In this transformation, the activated copper catalyst was proposed to react with the ligand, NaO*^t^* Bu, and B_2_pin_2_ to generate the (L)CuBpin complex, which adds to the alkene to form **INT‐10.1**. Subsequent CO insertion then delivers the acyl copper species **INT‐10.2**, which isomerizes to the carbene intermediate **INT‐10.3**. Carbene insertion into the β C─H bond gives the copper‐alkoxide cyclopropane species **INT‐10.4**. Another equivalent of B_2_pin_2_ afforded OBpin‐substituted species **INT‐10.5**, in which the OBpin acts as a leaving group to activate the C─O bond cleavage and furnish the desired diborylcyclopropanes **10.2** [76]. Notably, the authors did not discuss whether this OBpin–Bpin exchange at carbon proceeds with inversion or retention at the stereogenic center.

In order to overcome this protocol's limitation to terminal aliphatic olefins, the same group developed a copper‐catalyzed carbonylative cyclopropanation of aryl olefins in 2022 (Scheme 10b) [77]. The mechanism proceeded in a similar manner via the unusual in situ α‐oxy carbene intermediate, thereby substantially broadening the accessible substrate class. The reaction was optimal with CuCl and a DPPP ligand in fluorobenzene as solvent, and both the temperature (60°C) and CO pressure (30 bar) exerted a significant influence on the yield. The substrate scope encompassed a range of *para*‐, *ortho*‐, and *meta*‐substituted *trans*‐β‐methylstyrenes and vinylbenzenes (**10.3**), delivering 1,2‐diborylated cyclopropanes (**10.4**) in moderate to good yields. In contrast, internal aliphatic olefins and *cis*‐β‐methylstyrene failed to undergo cyclopropanation. In 2023, Wu et al. also expanded this methodology to access β‐boryl substituted cyclopropane structures [78].

Another inventive approach was reported by Zhu and coworkers in 2024, who developed a copper‐catalyzed method generating α‐boryl carbenes from (Bpin)‐substituted cyclopropenes (**10.5**) for highly selective cyclopropanation of alkenes, along with B─H and Si─H insertions (Scheme 10c) [44]. The Cu(MeCN)_4_PF_6_/chiral bisoxazoline ligand **L3** system proved effective for borylcyclopropanation, affording optically active cyclopropyl boronic esters (**10.7**) in high yields with excellent selectivities via an α‐boryl carbene intermediate **INT‐10.6**. A broad range of alkenes (**10.6**) was tolerated, including aryl‐, alkyl‐, functionalized, and 1,1‐disubstituted variants. Notably, the method enabled synthesis of previously elusive chiral 1,2‐diheteroatom‐substituted and tetrasubstituted cyclopropanes. They also developed a way to synthesize cycloheptadienyl boronic esters via rearrangement of an in situ borylcyclopropanes (see Section 7.4, Scheme 31h). Mechanistic studies identified Cu(I)‐mediated cyclopropene ring‐opening as the rate‐determining step. Computations revealed that the Bpin moiety both polarizes the cyclopropene double bond to dictate chemoselectivity in ring‐opening and imposes steric hindrance with a rigid, perpendicular orientation relative to the carbene plane, enabling the catalyst's chiral pocket to discriminate *Re*‐facial attack for stereocontrol.

#### 2.2.5 Cobalt Carbenoid Mediated Reactions

**SCHEME 11:**
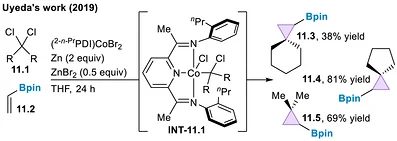
Cobalt carbenoid mediated reactions.

Werth and coworkers reported a cobalt pyridine diimine (PDI)‐catalyzed reductive spirocyclopropanation of terminal 1,3‐dienes using *gem*‐dichlorocycloalkanes (**11.1**) and Zn as terminal reductant (Scheme 11) [79]. A key advantage of this cobalt carbenoid system over classical Zn carbenoids was its resistance to 1,2‐hydride shifts, suppressing alkene byproduct formation that had previously hampered substituted carbenoid cyclopropanation. DFT studies suggested the active carbenoid intermediate **INT‐11**. is best described as a (PDI)Co(CR_2_)Cl_2_ complex. The authors found vinylBpin (**11.2**) to be a viable substrate under the standard conditions, delivering spirocyclic organoboron products **11.3–11.5** in moderate‐to‐good yields.

#### 2.2.6 Iron Carbene Mediated Reactions

**SCHEME 12:**
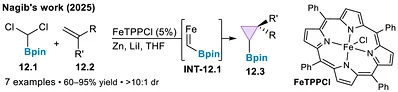
Iron carbene mediated reactions.

Nagib and coworkers reported an Fe‐catalyzed cyclopropanation strategy using commercially available *gem*‐dichlorides as carbene precursors, FeTPPCl as catalyst, and Zn as a reductant, enabling unified catalytic access to donor, neutral, and acceptor carbenes from a single platform (Scheme 12) [80]. The proposed mechanism proceeds via in situ halide exchange of the *gem*‐dichloride with LiI to generate a more easily reduced iodide, which undergoes SET reduction by Zn to form an α‐chloro radical, captured by Fe(II) to generate the reactive Fe‐carbene intermediate **INT‐12.1** [81]. Critically for borylcyclopropane synthesis, a Bpin‐substituted *gem*‐dichloride (**12.1**) was demonstrated as a competent neutral carbene precursor, delivering borylcyclopropanes **12.3** in good yields. The same Fe‐catalyzed platform was subsequently extended by the same group to deuterated cyclopropanation using CD_2_Cl_2_ as carbene precursor, achieving >99% deuterium incorporation without the H/D exchange issues associated with diazomethane‐d_2_ [81].

#### 2.2.7 Metal‐Free Carbene Mediated Reactions

**SCHEME 13:**
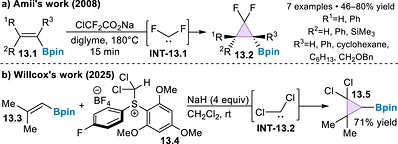
Metal‐free carbene mediated reactions via cyclopropanation of borylated alkenes.

In 2008, Fujioka and Amii developed a metal‐free, stereospecific synthesis of boron‐substituted *gem*‐difluorocyclopropanes **13.2** (Scheme 13a) [82]. These products were prepared via two protocols from vinylboronic acid pinacol esters **13.1**: (1) brief heating with sodium chlorodifluoroacetate in diglyme at 180°C for 15 min, or (2) syringe‐pump addition of a diglyme solution of the reagent over 8 h at 180°C, with the latter procedure delivering consistently superior yields. The method tolerated various 1‐substituted *E/Z* vinylboronic esters and a cyclic trisubstituted example was shown, with difluorocarbene (**INT‐13.1**) cycloadditions proceeding stereospecifically to preserve alkene geometry.

Utilizing a different free carbene precursor, Moore and Wilcox developed dichloromethyl(diaryl) sulfonium salts (**13.4**) as bench‐stable precursors for *gem*‐dichlorocyclopropanation of electron‐rich olefins via a free singlet dichlorocarbene pathway (Scheme 13b) [83], inspired by earlier reports on dihalomethyl‐substituted sulfonium salts [84, 85]. A broad range of electron‐rich olefins was converted to the corresponding *gem*‐dichlorocyclopropanes in moderate to good yields. Of particular relevance, the methodology demonstrated tolerance of the Bpin functionality, converting alkene **13.3** to pentasubstituted cyclopropane **13.5** via a dichlorocarbene (**INT‐13.2**). Although the desired product was formed in 71% NMR yield, purification difficulties resulted in unsuccessful isolation of the product, highlighting a remaining challenge for borylcyclopropane chemistry.

**SCHEME 14:**
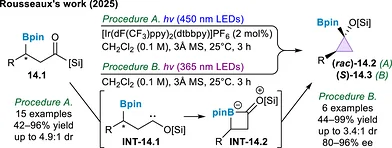
Metal‐free carbene mediated reactions via 1,2‐shift.

In 2025, Palermo and coworkers presented the synthesis of enantioenriched 1,1‐silyloxycyclopropylboronic esters from photoexcited β‐boryl acylsilanes (Scheme 14) [86]. Acyclic β‐boryl acylsilane precursors (**14.1**) were first photoexcited to generate α‐silyloxycarbenes (**INT‐14.1**) through a photochemical 1,2‐Brook rearrangement, followed by intramolecular cyclization to a four‐membered boronate (**INT‐14.2**). This intermediate then underwent a strain‐increasing 1,2‐boronate rearrangement that contracted the ring, delivering densely functionalized, stereodefined borylated cyclopropanols. Under optimized conditions, an iridium‐catalyzed, triplet energy transfer‐driven photocyclization at 450 nm afforded various alkyl‐ and aryl‐substituted siloxycyclopropylboronic esters (**14.2**) in high yields and modest diastereoselectivities. The pathway, however, was not suitable for preparing enantioenriched borylcyclopropanes because an unproductive EnT‐mediated racemization pathway. To circumvent this, enantioenriched acyclic β‐boryl acylsilanes were instead subjected to direct visible‐light excitation at 365 nm, thereby avoiding stereoablative processes and providing a series of enantioenriched cyclopropanol derivatives (**14.3**) that would otherwise be difficult to access.

## 3 Intramolecular Nucleophilic Ring Closure Routes to Borylcyclopropanes

### 3.1 Transition‐Metal‐Catalyzed Methods

**SCHEME 15:**
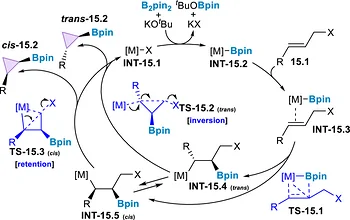
General transition‐metal catalyzed intramolecular borylcyclopropanation mechanism.

Several methods convert allylic electrophiles to borylcyclopropanes via an intramolecular cyclization (Scheme 15). These strategies are notable for the diversity of catalytic and non‐catalytic protocols, the wide range of participating substrates, and the ability to control both absolute and relative stereochemistry through a combination of substrate and reagent control. The transition‐metal catalyzed reactions typically initiate with the reaction between a metal salt (**INT‐15.1**) and a diboron reagent to give the active metal–boryl species (**INT‐15.2**). This intermediate reacts with the allyl electrophile (**15.1**) to form the π‐complex **INT‐15.3**. The borylmetalation of this intermediate via transition state **TS‐15.1** yields alkylmetal intermediates **INT‐15.4** or **INT‐15.5**. Subsequent intramolecular nucleophilic displacement of a leaving group (often the diastereoselectivity‐determining step) proceeds to generate cyclopropanes **15.2** via transition state **TS‐15.2** or **TS‐15.3**. Product stereochemistry is dictated by (a) the facial selectivity of the olefin addition, (b) the olefin configuration, and (c) whether nucleophilic substitution proceeds with inversion or retention of stereochemistry of the C─M bond in the ring‐closure step.

**SCHEME 16:**
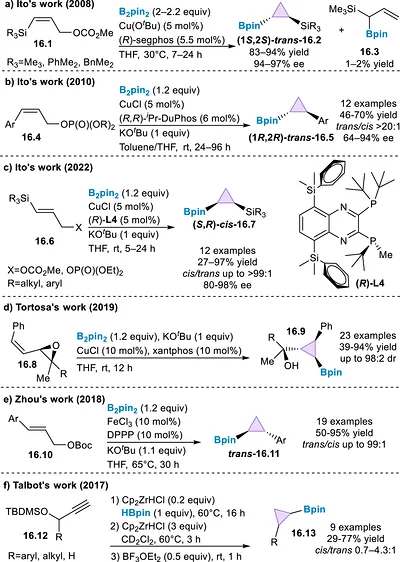
Transition‐metal catalyzed intramolecular nucleophilic ring closure routes to borylcyclopropanes.

In 2008, Ito et al. unexpectedly formed boryl‐silylcyclopropane **16.2** (along with trace amounts of allylboron **16.3**) while attempting to synthesize enantioenriched allylboronic esters with copper(I) catalysis (Scheme 16a) [87]. They found that cyclopropanes were formed significantly faster from silyl‐substituted (*Z*)‐allylic carbonates **16.1** compared to alkyl‐substituted allylic carbonates, implying the silyl group had an activation role. Although (*E*)‐allylic carbonates were viable, they required significantly longer reaction times and gave mixtures of *trans* and *cis* diastereomers alongside the allylboronic ester, prompting the authors to focus instead on the more selective (*Z*)‐substrates. With unoptimized conditions already delivering 99:1 *trans/cis* selectivity, the authors optimized enantioselectivity for (1*S*,2*S*)‐*trans*‐silyl‐borylcyclopropanes through screening of chiral phosphine ligands and systematic variation of the silicon‐bound alkyl substituents.

The same group in 2010 reported the enantioselective copper‐catalyzed synthesis of *trans*‐aryl‐ and ‐heteroaryl‐substituted borylcyclopropanes (**16.5**) from (*Z*)‐allylic phosphates **16.4**, using (*R,R*)‐*^i^* Pr‐DuPhos as the chiral ligand (Scheme 16b) [88]. With optimized reaction conditions, they found that a range of substituents on the benzene ring were supported, with electron‐donating substituents resulting in higher enantioselectivity than electron‐withdrawing groups. The reaction also supported various aromatic heterocyclic groups such as indole and thiazole, but alkenyl substituents provided no product. When examining the corresponding (*E*)‐allylic phosphates, the authors curiously found a diastereoselectivity switch dependent on catalyst. With (*R,R*)‐*^i^* Pr‐DuPhos, (*E*)‐allylic phosphates formed *cis*‐borylcyclopropanes with high diastereoselectivity, but modest ee. In contrast, with (*R,R*)‐QuinoxP*, *E* substrates predominantly gave *trans*‐borylcyclopropanes with complete diastereoselectivity but poor enantioselectivity. The authors proposed that both reactions proceed through boryl cupration, with ring‐closure proceeding with retention of the C─Cu stereochemistry when (*R,R*)‐*^i^* Pr‐DuPhos was used and inversion with (*R,R*)‐QuinoxP* as the ligand.

In 2022, Ito and coworkers extended their 2008 work by developing a chiral bisphosphine ligand to access the thermodynamically disfavored (1*S*,2*R*)‐*cis*‐silyl‐borylcyclopropanes, supported by computational studies on enantio‐ and product‐selectivities (Scheme 16c) [89]. The authors identified (*E*)‐allylic phosphates (**16.6**) as superior to carbonates for *cis* selectivity. Ligand screening identified (*R*)‐Quinox‐*^t^* Bu_3_ as a promising lead (96:4 *cis/trans*, 89:11 borylcyclopropane:allylboronic ester). To further enhance stereocontrol, bulky silyl groups were installed on the ligand backbone; x‐ray crystal structures confirmed interlocking of the silane and phosphine moieties, and the DMPS‐modified (*R*)‐5,8‐*Si*‐Quinox‐*^t^* Bu_3_ (**L4**) furnished (*S,R*)‐*cis*‐silyl‐borylcyclopropanes (**16.7**) from (*E*)‐allylic phosphates in excellent yield (98%), enantioselectivity (96% ee), diastereoselectivity (>99:1 *cis/trans*), and chemoselectivity (99:1 borylcyclopropane:allylboronic ester). γ‐Silylated (*E*)‐allylic phosphates bearing diverse alkyl and aryl groups on silicon were well tolerated, delivering bifunctional boron–silicon cyclopropanes with consistently high stereocontrol. Computational analysis revealed how silyl modifications of the ligand rigidified the phosphine environment to enhance regio‐ and enantioselectivities, with DFT calculations suggesting stereoretentive intramolecular cyclization via nucleophilic attack at the stereogenic copper‐bound carbon.

Exploiting vinyl epoxides as electrophilic partners, Tortosa and coworkers in 2019 reported an enantioselective, stereospecific synthesis of α‐hydroxy borylcyclopropanes, in which copper(I) catalyzed borylation of enantiomerically enriched vinyl epoxides (**16.8**) was followed by an intramolecular cyclization to furnish products bearing four contiguous stereocenters (Scheme 16d) [90, 91]. In optimizing the reaction, the authors found that increasing the bite angle of the phosphine ligand favored borylcyclopropane formation over the diene side product formed by deborylation and ring‐opening of the epoxide, followed by β‐oxygen elimination. Only the combination of xantphos and KO*^t^* Bu base (rather than LiO*^t^* Bu) suppressed the diene formation and resulted in excellent reactivity for trisubstituted α‐hydroxy borylcyclopropanes (**16.9**). Single‐crystal x‐ray crystallography determined the relative configuration of the four contiguous stereocenters. Evaluation of the scope at the aryl group on these borylcyclopropanes revealed good tolerance to various stereoelectronic effects on the *ortho*‐, *para*‐, and *meta*‐ positions. Additionally, excellent chirality transfer was observed, as changes in the geometry of the epoxide and double bond were found to create different diastereomers with control over the four stereocenters without modifying the reaction conditions. For instance, *E*‐alkene gave access to borylcyclopropanes with a *trans* relationship between the aryl group and the boron atom. The authors note that this literature represents the first reported cyclopropanation via a copper‐catalyzed borylative cyclization involving a secondary sp^3^–electrophile.

Iron catalysis offered an unexpected alternative for this transformation, with Zhou and coworkers demonstrating an iron‐catalyzed, stereoselective synthesis of disubstituted aryl borylcyclopropanes (**16.11**) from cinnamyl carbonates (**16.10**) in 2018 (Scheme 16e) [92]. Under ligand‐free conditions, several metal salts were evaluated, but FeCl_3_ provided the highest yields and selectivities, whereas other transition‐metal chlorides such as CuCl, CoCl_2_, ZnCl_2_, CrCl_3_, and PdCl_2_ gave low yields or poor diastereocontrol. Introducing ligands had little impact on reactivity; they mainly enhanced stereoselectivity at the cost of longer reaction times. A range of para‐substituted aryl groups bearing electron‐donating or electron‐withdrawing substituents, as well as larger polycyclic aromatics, were well tolerated, while heteroaryl and alkyl substrates failed to produce product. Overall, the method delivered the borylcyclopropanes in moderate‐to‐good yields with excellent diastereoselectivity, favoring the *trans* diastereomer. Compared to the 2010 report by Ito and coworkers [88], this iron‐catalyzed approach affords similar diastereoselectivity (99:1 vs. >20:1 *trans/cis*) and similar yields, though the authors demonstrate complementary substrate profiles: copper catalysis accommodates a furanyl substituent while iron catalysis uniquely delivers a dioxaborinane‐substituted product.

In 2017, Talbot and coworkers reported a zirconium‐based methodology to convert propargylic alcohols **16.12** to borylcyclopropanes **16.13** (Scheme 16f) [93]. Building on the cyclopropanation method developed by Gandon and Szymoniak in 2002 [94], the authors carried out a Schwartz's reagent‐catalyzed hydroboration, with subsequent hydrozirconation followed by Lewis's acid‐mediated cyclization. Various leaving groups were examined, including methyl, benzyl, and silyl ether systems, with the silyl propargyl ethers giving the highest proportions of borylcyclopropanes. The diastereomeric ratio was found to depend on the size of the silyl protecting group: the bulkier tris(trimethylsilyl) group favored the *cis* diastereomer (3.8:1), whereas the smaller triethoxysilyl group predominantly afforded the trans diastereomer (0.4:1). The reaction scope was restricted to disubstituted borylcyclopropanes bearing benzene rings with varied electronic properties and alkyl substituents, which were obtained in moderate yields, along with a single trisubstituted example in low yield. In contrast, substrates containing heteroaryl or spirocyclic substituents did not react successfully. Notably, the zirconium‐mediated reaction is mechanistically complementary to the copper‐catalyzed and iron‐catalyzed approaches of Ito [88] and Zhou [92] respectively (hydrometalation vs. boryl‐metalation). With regard to synthetic utility, this zirconium‐based method accommodated aliphatic substituents such as ethylphenyl and ethylfuranyl groups that fall outside the scope of those aryl‐ and heteroaryl‐limited conditions, although diastereoselectivities were generally modest.

### 3.2 Transition‐Metal Free Methods Employing Geminal Diborylalkanes

**SCHEME 17:**
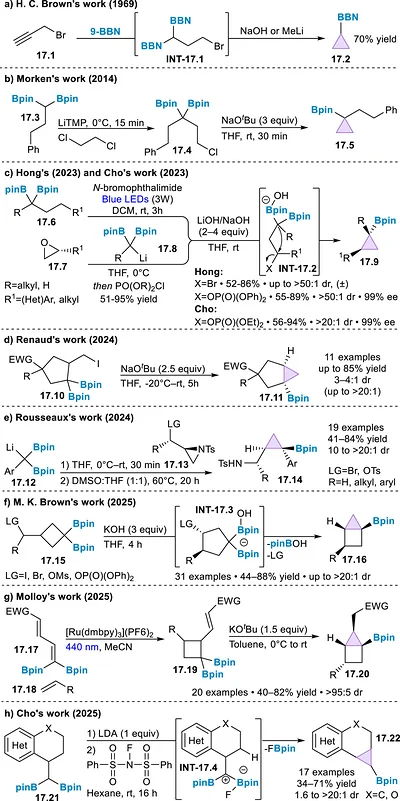
Transition‐metal free, deborylative methods employing *gem*‐diborylalkanes.

The first nucleophilic route to a borylcyclopropane dates to 1962, when Binger and Köster synthesized diethylcyclopropylborane via the dihydroboration of propargyl chloride and diethylborane [95]. Seven years later, H. C. Brown and coworkers were able to realize this product (**17.2**) under the presence of aqueous hydroxide or methyllithium at room temperature, as opposed to 130°C (Scheme 17a) [96]. In 1966, Matteson and Schaumberg synthesized dibutyl 2,2‐dicyanocyclopropaneboronic ester via base‐mediated intramolecular cyclization, yet this early finding remained largely unexplored for decades [97]. Recent years have seen a resurgence of interest in transition‐metal‐free cyclization strategies, with *gem*‐diborylalkanes emerging as particularly powerful reagents: the electron‐withdrawing effect of two adjacent boron atoms renders the α‐carbon sufficiently acidic to generate a stabilized carbanion upon deprotonation, enabling borylcyclopropanation without any transition metal catalyst.

In 2014, Morken coworkers developed an efficient route to cyclic organoboronic esters through α‐boryl carbanions [12]. The authors discovered that deborylative alkylation of *gem*‐diborylalkanes was best achieved by alkoxide bases under anhydrous conditions rather than with hydroxide bases. In addition to utilizing this procedure to synthesize hindered secondary and tertiary boronic esters, they also reported an intramolecular ring‐forming deborylative alkylation with diboronic ester **17.4** that produced borylcyclopropane **17.5** (Scheme 17b). In 2023, Hong and coworkers developed efficient methods to convert readily available *gem*‐diborylalkanes into both racemic and enantioenriched borylcyclopropanes (Scheme 17c) [16]. For the racemic variant, Wohl–Ziegler benzylic bromination of **17.6** was followed by deborylative cyclization with LiOH·H_2_O in a one‐pot fashion, delivering *trans*‐borylcyclopropanes **17.9** via intermediate **INT‐17.2** in 51%–95% yield with >50:1 dr (except with bulky α‐alkyl substituents). The R^1^ scope was accomodated a range of (hetero)aryl and alkyl groups. The enantioselective route used ring‐opening of optically active epoxides (**17.7**) with lithiated diborylalkanes **17.8**, followed by phosphorylation to form chiral phosphate‐bearing diborons, and LiOH·H_2_O‐mediated cyclization via intermediate **INT‐17.2** to give enantioenriched cyclopropylboronic esters (**17.9**); this method was higher yielding over two steps compared to the racemic variant. Here the R group α to boron remained restricted to alkyl, but R' expanded to diverse alkyl chains alongside aryl and heteroaryl groups, with >99% enantiospecificity across all examples. Notably, LiOH·H_2_O outperformed NaO*^t^* Bu (good yield but low dr), contrasting to Morken's 2014 report in which hydroxide triggered *trans*‐metalation to Pd or protodeboronation but was incapable promoting alkylation with alkyl halides. The authors proposed a concerted 1,3‐elimination mechanism with inversion of stereochemistry at both the boron‐bearing carbon and the electrophilic carbon.

Two months later, Cho and coworkers published a very similar route to enantioenriched borylcyclopropanes, using diethyl phosphate instead of diphenyl phosphate and NaOH instead of LiOH [98]. The authors demonstrated R^1^ (**17.7**) groups including substituted phenyl, benzyl, and dihydrocinnamyl. The substrate scope of R (**17.6**), however, was explored in further depth by Cho's group and found to accomodate larger and more complex substituents such as lithocholic acid with high yields and excellent stereoselectivities. Adding to the breadth of the deborylative alkylative routes, the Hong group extended their 2023 study to incorporate the diastereoselective synthesis of boryl‐substituted vinylcyclopropanes in 2025 [18]. Using a similar approach, Renaud et al. performed a deborylative alkylation to synthesize fused bicyclic cyclopropylboronic esters in 2024 (Scheme 17d) [99]. Many terpenoid natural products contain the bicyclo[3.1.0]hexane skeleton monoalkylated at the bridgehead position. The authors first prepared γ‐iodo‐*gem*‐diborylated cyclopentanes **17.10** via a radical annulation of *gem*‐diborylethene with various iodoalkanes using Et_3_B and di‐*tert*‐butylhyponitrite (DTBHN) as initiators. Subsequent treatment of these gem diborylated cyclopentanes with an alkoxide base furnished borylated bicyclo[3.1.0]hexanes (**17.11**) in good yields.

Expanding these concepts to aziridines, a 2024 study by Rousseaux and coworkers reported the diastereoselective synthesis of borylated (aminomethyl)cyclopropanes (**17.14**) using lithiated *gem*‐diborylalkanes **17.12** and α‐halo and α‐tosyloxy aziridines **17.13** (Scheme 17e) [17]. The reaction tolerated 1‐aryl substituted 1,1‐diborylalkanes with substituents at the *ortho*‐, *meta*‐, and *para*‐ positions. Heteroaromatic groups like pyrrole and furan were tolerated as substituents on the aromatic ring, but *gem*‐diborylalkanes that were non‐substituted, alkyl‐substituted, or substituted with sterically hindered arenes like mesityl were not successful. The authors proposed that the mechanism proceeded with ring‐opening of aziridine by lithiated *gem*‐diborylalkane, resulting in a sulfonamide (detected by ^1^H NMR), which performed an intramolecular S_N_2 reaction to form another aziridine. This step was thought to be facilitated by polar aprotic solvent, which was found to be crucial for product formation. Finally, Lewis's base‐mediated deborylation and diastereoselective 3‐exo‐tet cyclization opened the aziridine ring to form the borylated (aminomethyl)cyclopropane. DFT calculations were used to rationalize the relationship between aziridine stereochemistry and reaction diastereoselectivity.

In 2025, Brown and coworkers reported an unexpected synthesis of borylated housanes (**17.16**) via an intramolecular deborylative alkylation of *gem*‐diboryl cyclobutanes (**17.15**), themselves prepared by strain‐release diboration of bicyclo[1.1.0]butane (Scheme 17f) [100]. Notably, the target BCP product was not observed under any conditions, with the housane arising instead as the sole product. The reaction proceeded via an unusual boron‐enabled ring expansion through a cyclopentane intermediate (**INT‐17.3**), proposed to occur via an asynchronous concerted dyotropic rearrangement; computational NBO analysis revealed significant electronic communication between the C─borate bond and a three‐center two‐electron interaction in the transition state, identifying borate formation as the critical driving force for ring expansion. KOH was identified as optimal activator, and a single diastereomer was observed across a broad scope of aryl and alkyl substituents, with diastereoselectivity rationalized by an antiperiplanar arrangement of the cyclobutane C─C bond relative to the leaving group in the reactive conformation.

Shortly after, Molloy and coworkers reported a complementary boron‐enabled strategy for the synthesis of polysubstituted housanes (**17.20**) from nonsymmetrical diene precursors (**17.17**) in just two steps (Scheme 17g) [101]. A geminal diboron system proved critical for establishing complete site‐ and regioselectivity in an energy transfer‐catalyzed intermolecular [2+2] cycloaddition, since alternative boron moieties including BPin, BMIDA, and BF_3_K all afforded complex mixtures of dimers and isomers, attributed to insufficient electronic bias at the diene termini. Subsequent KO*^t^* Bu‐mediated deborylative intramolecular conjugate addition delivered the housane products as single diastereomers. DFT studies revealed that *tert*‐butoxide preferentially attacks the less sterically encumbered BPin unit, generating a stabilized borata‐alkene‐like carbanion intermediate, which then undergoes stereospecific ring closure, with the observed diastereoselectivity arising from steric differentiation between phenyl and acrylic residues in the two possible carbanion rotamers.

Departing from carbanion‐based reactivity, Cho and coworkers unveiled a strategic fluorine‐triggered cyclopropanation from *gem*‐diborylalkanes **17.21** in 2025 (Scheme 17h) [23]. Their approach leveraged [1, 3]‐hydride shifts in transient α‐boryl cations generated from α‐fluoro‐diborylalkanes. The authors propose that treatment of the diboryl‐substituted anion with *N*‐ fluorobenzenesulfonimide effected α‐fluorination. Then, [1, 2]‐fluoride migration generated an α‐boryl cation (**INT‐17.4**) that selectively underwent a [1, 3]‐hydride shift via a three‐center two‐electron interaction, enabling efficient synthesis of cyclopropyl boronic esters (**17.22**). Computational and mechanistic studies elucidated the key electronic factors driving this unusual hydride shift mechanism and discounted an alternative mechanism involve an α‐boryl carbene.

**SCHEME 18:**
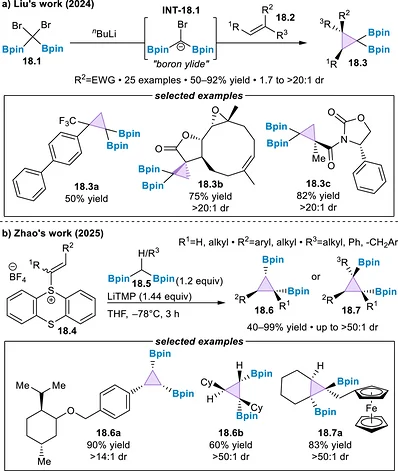
Transition‐metal free, non‐deborylative methods employing *gem*‐diborylalkanes.

Moving away from the deborylative alkylating routes but building on the chemistry of α‐halo *gem*‐diborylalkanes [102, 103], Liu and coworkers demonstrated the stereoselective construction of *gem*‐diborylcyclopropanes (**18.3**) via boron‐stabilized carbanion intermediates they describe as “boron ylides” (Scheme 18a) [21]. Whereas an ylide carbanion is stabilized by a positively charged heteroatom which also serves as the leaving group in cyclopropanations (e.g., Corey–Chaykovsky), the authors of this study envisioned separating the two functions by stabilizing the α‐carbanion by the two vacant p‐orbitals of the boryl groups instead. First, diborodibromomethane (DBDBM) was synthesized by the migrative diborylation of tribromomethyllithium. DBDBM then underwent lithium‐halogen exchange to form the “boron ylide” (**INT‐18.1**), followed by reaction with an electron‐deficient olefin (**18.2**) to form *gem*‐diborylcyclopropanes in excellent yield and diastereoselectivity (>20:1 dr). The substrate scope is the most diverse of the publications in this category, allowing for ester, amide, cyano, sulfonyl, phosphonyl, fluoroalkyl (**18.3a**), amine, and even complex structures like parthenolide (**18.3b**). The reaction was unsuccessful with α‐methylstyrene and 1‐octene, limiting the method to electron‐deficient olefins, and contrasting with the majority of borylcyclopropanation methods reported in the literature, which predominantly accommodate aryl‐ and alkyl‐substituted alkenes. Chiral auxiliaries were incorporated to facilitate asymmetric induction, and it was found that the phenyl‐substituted oxazolidone auxiliary (**18.3c**) afforded the products in excellent diastereoselectivity (>20:1 dr). Furthermore, the *^t^* butyl‐substituted oxazolidone auxiliary lead to an excellent enantioselectivity after alcoholysis and aminolysis. Mechanistic studies indicated that the reason for this was the pre‐coordination of the carbonyl oxygen with boron prior to the nucleophilic attack of “boron ylide” to generate a six‐membered enolate intermediate, followed by intramolecular cyclization to yield *gem*‐diborylcyclopropane. As this Fang et al. study [21] was being published, the Hong group was working on a similar approach [20]. While the Liu group's methodology focused on α,β‐unsaturated esters, amides and their analogs, they expanded their scope to *gem*‐diborylcyclopropyl ketones through conjugate addition of lithiated chlorodiborylmethane to α,β‐unsaturated ketones.

In 2025, Zhao and coworkers reported an efficient diastereoselective synthesis of 1,2‐diboronic ester‐substituted cyclopropanes (**18.6** and **18.7**) from *gem*‐diborylalkanes (**18.5**) and alkenylthianthrenium salts (**18.4**) via a 1,2‐boronate rearrangement (Scheme 18b) [19]. The thianthrene group serves a dual role: activating the alkene toward nucleophilic addition by the *gem*‐diborylalkane carbanion while simultaneously functioning as a leaving group to trigger 1,2‐migration of the tetra‐coordinated boron species, forging the cyclopropane ring. Optimization identified Li‐TMP as the optimal base in THF at −78°C; commercially available bases such as LiHMDS, NaHMDS, and KHMDS gave only trace product, while LDA provided moderate yield but lower diastereoselectivity. The substrate scope was broad, encompassing styrenyl thianthrenium salts bearing electron‐donating and electron‐withdrawing substituents at *ortho*‐, *meta*‐, and *para*‐ positions, as well as aliphatic terminal and internal alkenes (**18.6b**), cyclic alkenes ranging from cyclopentene to cyclododecene, and complex natural product‐derived substrates like (−)‐menthol (**18.6a**), generally delivering good yields and high diastereoselectivities (14:1 to >50:1 dr). The *gem*‐diborylalkane component also tolerated diverse functional groups including alkyl, aryl, heteroaryl, silyl ether, benzyl, halogen, ferrocene (**18.7a**), and ketal substituents, though yields were more variable (moderate‐to‐excellent), with diastereoselectivity generally maintained across the series. The gram‐scale reaction proceeded in 70% yield and 15:1 dr, confirming the practical scalability of the method.

### 3.3 Radical Pathways to Borylcyclopropanes

Photocatalytic cyclopropanation has emerged as a powerful and mild alternative to carbene‐based and other transition‐metal‐based approaches, harnessing visible light to generate reactive radical intermediates under operationally simple conditions. The application of this strategy to borylcyclopropane synthesis has recently been explored, with α‐ and β‐boryl radicals serving as key intermediates whose unique stabilization by the adjacent boron center enables selective cyclopropanation pathways distinct from those accessible through ionic or carbene‐based chemistry.

**SCHEME 19:**
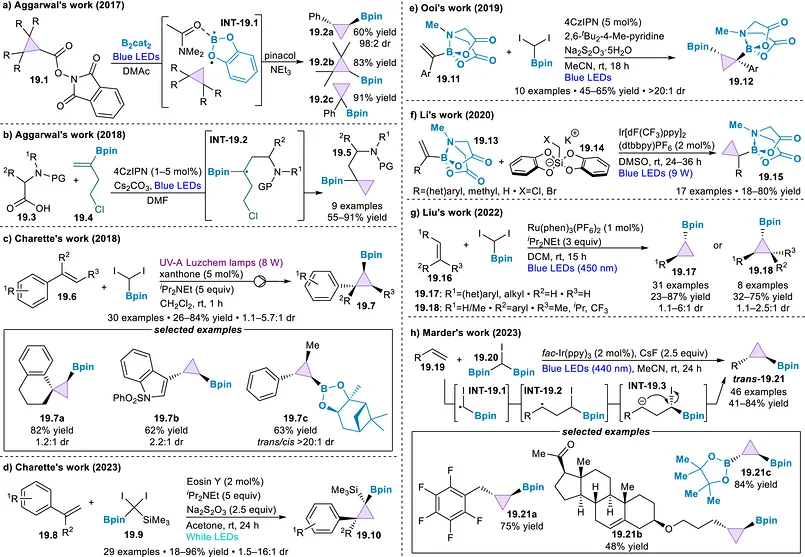
Radical pathways to borylcyclopropanes.

In 2017, Fawcett, Aggarwal and coworkers reported a photoinduced decarboxylative borylation of *N*‐hydroxyphthalimide esters using bis(catecholato)diboron under blue LED irradiation at ambient temperature, proceeding via a boryl radical chain mechanism (Scheme 19a) [104]. Within the substrate scope, cyclopropane‐containing redox‐active esters (**19.1**) were successfully converted to the corresponding pinacol boronic esters (**19.2a–c**). Notably, the 1,2‐disubstituted cyclopropane product (**19.2a**) was obtained with high diastereoselectivity (98:2 dr). The reaction was proposed to proceed via decarboxylation of the N‐hydroxyphthalimide ester to generate an alkyl radical, which combined with a DMAc‐stabilized boryl radical (**INT‐19.1**) derived from B_2_cat_2_ to generate the desired boronic ester. Shortly after in 2021, Barton and coworkers reported an electrochemical variant of the same decarboxylative borylation strategy, again proceeding through redox‐active ester intermediates, and applied it to a formal synthesis of the polycyclopropane natural product, jawsamycin [105].

Aggarwal and coworkers also presented a radical addition–polar cyclization cascade that provided a modular entry to functionalized cyclopropanes directly from carboxylic acids in 2018 (Scheme 19b) [106]. This platform was readily adaptable to form cyclopropyl boronic esters (**19.5**) in good‐to‐excellent yields via the reaction of aliphatic carboxylic acids (**19.3**) and haloalkyl alkenyl boronic esters (**19.4**). In their proposed mechanism, visible light photoredox catalysis enabled decarboxylation to an α‐amino radical. Conjugate addition to the electron‐poor alkene then formed a stabilized α‐boryl radical (**INT‐19.2**). Subsequent reduction to a carbanion preceded intramolecular S_N_2‐type cyclization to forge the cyclopropane. The methodology tolerates a wide range of functional groups on both coupling partners, including complex amino acids, dipeptides, and densely functionalized natural products.

A distinct photochemical strategy was employed by Charette and coworkers in 2018, who harnessed UV light rather than visible‐light photoredox catalysis to achieve borylcyclopropanation of styrenes under continuous flow conditions, using a custom photoreactor in which FEP tubing was wrapped around four 8 W UV A (350 nm) Luzchem lamps to ensure efficient and uniform irradiation (Scheme 19c) [107]. Exploiting the lability of the C─I bond, diiodomethylboronic acid pinacol ester was subjected to UV A irradiation in the presence of styrene and Hünig's base, initially giving the desired aryl borylcyclopropane in 42% yield. Introduction of a photosensitizer (5 mol% xanthone) reduced the residence time from 3 h to 1 h while increasing the yield to 78%. The method displayed a relatively broad scope: electron‐rich and electron‐poor styrenes, heteroaryl substrates, and spiro bicyclic alkenes (**19.6**) all afforded the corresponding borylcyclopropanes (**19.7**) in moderate‐to‐good yields and moderate diastereoselectivities. High reactivity was generally limited to terminal alkenes; however, switching from diiodomethylboronic acid pinacol ester to the corresponding pinanediol ester improved performance for internal and more highly substituted styrenes, boosting yields of resulting borylcyclopropanes (**19.7c**) by about 20%. Under UV A irradiation, xanthone was proposed to be promoted to its triplet excited state, which could be quenched either reductively by Hünig's base or oxidatively by diiodomethylboronic acid pinacol ester. Stern–Volmer experiments indicated that both processes were viable and might operate in parallel, although the authors proposed that the reductive quenching pathway predominated. In this pathway, the resulting xanthone radical anion (X·^−^), a strong reductant, transfers an electron to the diiodomethylboronic acid pinacol ester to generate a transient radical anion that fragments to an iodomethyl pinacol boronate radical, which then adds to styrene to furnish a benzylic radical. In this paper, the authors proposed S_H_2‐type cyclization with loss of iodine radical to provide the observed borylcyclopropanes, although a later paper proposed an alternative final step (see below).

In 2023, the same group reported the first synthesis of 1,1,2‑tri‑ and 1,1,2,2‑tetrasubstituted *gem*‑borosilylcyclopropanes (**19.10**) under visible‑light irradiation (Scheme 19d) [31]. Organocatalyzed by eosin Y, the reaction of diiodoboromethylsilane (**19.9**, prepared in a four‑step sequence) with styrene (**19.8**) derivatives in the presence of *^i^* Pr_2_NEt, sodium thiosulfate, and white LEDs afforded the desired products. The substrate scope closely resembled that of their 2018 work, but now with a preference for the diastereomer with the aryl group *cis* to the boronic ester. Notably, the scope included late‑stage functionalization of several bioactive molecules. Computational studies indicated that the radical pathway has a substantially higher barrier than an anionic pathway involving single‑electron transfer to generate a benzylic anion prior to cyclization, making this anionic route the dominant mechanism. Building on the Charette group's methodology, Ooi et al. developed a related diastereoselective protocol using visible light irradiation (blue LED) in 2019 for the synthesis of doubly borylated cyclopropanes (**19.12**) from α‐MIDA boryl styrenes **19.11** (Scheme 19e) [33]. The resulting diborylcyclopropanes are highly versatile intermediates that could be selectively functionalized at each boron site, enabling diverse downstream transformations.

Adapting the Ooi group's methodology, Li and coworkers the following year employed alkenyl boronic acid MIDA esters (**19.13**) and a chloromethyl silicate **19.14** (in place of diiodomethylboronic acid pinacol ester) under iridium photoredox catalysis to access *gem*‐disubstituted borylcyclopropanes **19.15** (Scheme 19f) [32]. This strategy broadened the substrate scope beyond styrene‐derived substrates, enabling incorporation of a heteroaryl substituent as well as simple unactivated alkenes such as propene and ethylene, albeit in low yield for the latter two. Two years later, Liu and coworkers reported a similar visible light‐mediated, ruthenium photoredox catalyzed borylcyclopropanation of alkenes (**19.16**), which broadened the scope to include alkyl and spirocyclic substituents and enabled access to more highly substituted borylcyclopropanes (**19.17** and **19.18**) than earlier related methods (Scheme 19g) [29]. Interestingly, the cyclopropanation proceeded even in the absence of the photocatalyst, albeit at a reduced 45% yield for their test substrate. The authors suggest that I_2_CHB(pin) could be reduced by either an electron‐donor acceptor complex with ^i^Pr_2_NEt or by the reduced photocatalyst. Similarly, they indicate the possibility of both S_H_2 and radical‐polar crossover mechanisms for ring closure.

In 2023, moving away from diiodomethylboronic acid pinacol ester, Marder and coworkers adopted the more stable and easily prepared (diborylmethyl)iodide reagent CHI(Bpin)_2_ (**19.20**), enabling a broadly functional‐group‐tolerant borylcyclopropanation of alkenes (**19.19**) with operational simplicity and excellent diastereoselectivity (Scheme 19h) [30]. The optimized conditions employed *fac*‐Ir(ppy)_3_ photocatalyst, CsF base, and 440 nm blue‐LED irradiation in MeCN at room temperature for 24 h. This protocol dramatically expanded the alkene scope beyond the Liu group's methyl/isopropyl limitation to include distal esters, thioaryls, heterocycles, halides (**19.21a**), amides, phthalimides, sulfonamides, silyl groups, vinylboronates (**19.21c**), vinylsilanes, and complex motifs like pregnenolone (**19.21b**) and vitamin E derivatives (41%–84% yield). Styrenes, vinyl nitriles, vinyl esters, and trisubstituted alkenes remained unreactive. Mechanistic studies via Stern–Volmer analysis and radical trapping supported a reductive quenching pathway: CsF‐enabled reduction of the Ir* photocatalyst by CHI(Bpin)_2_ to generate a CHI(Bpin) α‐boryl radical (**INT‐19.1**) that adds to the alkene to form radical intermediate **INT‐19.2**. Subsequent SET and carbanion cyclization of **INT‐19.3** to the *trans*‐borylcyclopropane (**19.21**).

**SCHEME 20:**
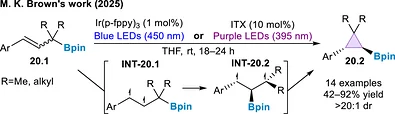
Radical pathways to borylcyclopropanes via a 1,3 biradical precursor.

In 2025, Brown and coworkers proposed accessing borylcyclopropanes via a 1,3 biradical precursor, in contrast to the [2+1] cycloaddition type strategies employed in the aforementioned studies (Scheme 20) [34]. The reaction begins with excitation of the starting alkene substrate (**20.1**) to a triplet biradical (**INT‐20.1**) via an energy transfer event, after which a 1,2‐boron migration generates a triplet 1,3 β‐boryl biradical intermediate (**INT‐20.2**) that collapses to the borylcyclopropane (**20.2**) through radical recombination coupled to intersystem crossing. The authors identified two effective photocatalytic conditions: one employing Ir(p‐fppy)_3_ and the other using the significantly less expensive photosensitizer isopropylthioxanthone (ITX), irradiated at 450 and 395 nm, respectively. Under these conditions, a range of aryl‐ and heteroaryl substituted alkenes furnished tetrasubstituted *gem* dimethylborylcyclopropanes in good yields and excellent diastereoselectivity (>20:1 dr), although heteroaryl substrates gave only moderate yields. Ene‐ynes were also competent substrates, providing the corresponding products in good yields but with somewhat reduced diastereoselectivity (4:1 dr).

## 4 C─H Activation Strategies for Cyclopropane Borylation

**SCHEME 21:**
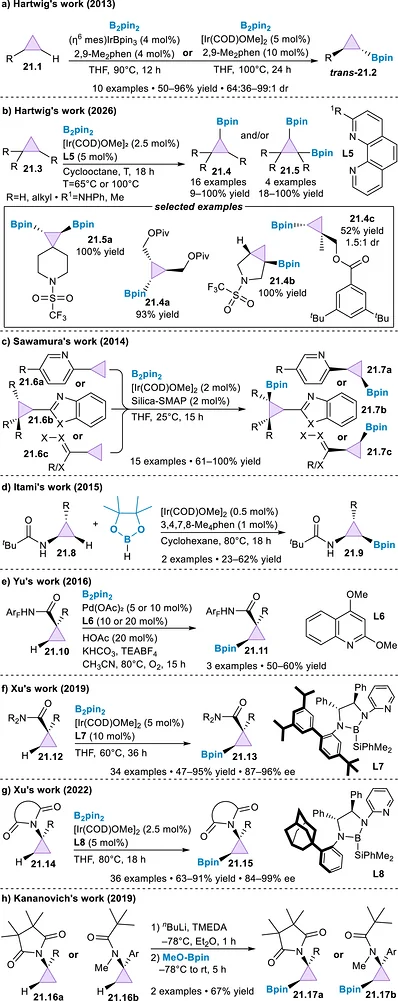
C─H activation strategies for cyclopropane borylation.

C─H activation represents one of the most atom‐economical strategies in modern organic synthesis, enabling direct functionalization of otherwise inert carbon–hydrogen bonds without pre‐functionalization. Its application to cyclopropane borylation offers a streamlined route to borylcyclopropanes from simple cyclopropane feedstocks, bypassing the need for alkene substrates or carbene precursors altogether. The first borylation of cyclopropane C─H bonds was reported by Hartwig and co‐workers in 2013, using an iridium–phenanthroline catalyst to access *trans*‐substituted borylcyclopropanes (**21.2**) without a directing group (Scheme 21a) [108]. Catalyst selection was guided by prior studies of iridium–trisboryl complexes in aromatic C─H functionalization [109, 110, 111], leading to the choice of (η^6^ mes)IrBpin_3_. During reaction optimization, the sterically hindered chelating nitrogen ligand 2,9‐Me_2_phen provided the highest yield and diastereoselectivity, with THF and B_2_pin_2_ identified as the optimal solvent and boron source, respectively. The substrate scope encompassed monosubstituted cyclopropanes (**21.1**) bearing halide, ester, carbonyl, nitrile, protected carbonyl, alcohol, and amine groups. Alkyl substituted cyclopropanes were less reactive, but their performance improved when cyclohexane was used as solvent and an excess of cyclopropane was added relative to the diboron reagent. Representative 1,1 and *cis*‐1,2 disubstituted cyclopropanes also underwent efficient borylation. Finally, the commercially available [Ir(COD)OMe]_2_ served as an alternative pre‐catalyst, affording moderate yields of borylcyclopropanes at 10 mol% loading and 100°C.

In 2024, the Hartwig group introduced 2‐aminophenanthroline ligands (**L5**) that enable iridium‐catalyzed, undirected C─H borylation of various alkyl groups under mild conditions [112]. Among the substrates tested was a single ether‐monosubstituted cyclopropane, which afforded the trans‐borylated product in moderate yield. Unlike their 2013 report, this method employs excess B_2_pin_2_ rather than excess cyclopropane. In early 2026, the same group further explored undirected C─H borylation of cyclopropanes (**21.3**), expanding the scope from mono‐ or disubstituted cyclopropanes to multisubstituted, fused, and spirocyclic classes of cyclopropanes (**21.4** and **21.5**) using their phenanthroline ligands under iridium catalysis (Scheme 21b) [113]. The optimized conditions depended on the cyclopropane class, with the ligand (2‐anilinophen or 2‐mphen) and temperature (65°C–100°C) adjusted accordingly. Vicinal (1,2‐substituted) *trans*‐ (**21.4a**) and *cis*‐disubstituted cyclopropanes were borylated in high yields. Spirocyclic cyclopropanes underwent borylation in moderate to high yields, forming mono‐ or diborylcyclopropanes in variable or, in some cases, fully selective ratios. For instance, a spirocyclic cyclopropane with a distal triflamide was converted to a diborylcyclopropane (**21.5a**) in quantitative yield and complete product selectivity with 2‐anilinophen at 100°C. This excellent degree of borylation was attributed to the electron‐withdrawing triflyl group activating the cyclopropyl C─H bonds toward a second borylation through a long‐range inductive effect. By comparison, the corresponding *N*‐Boc‐piperidine cyclopropane derivative gave both mono‐ and diborylated products in 46% and 15% yield, respectively. Non‐spirocyclic 1,1‐disubstituted cyclopropanes gave both diastereomers of the monoborylated product (**21.4c**), with a slight preference for the isomer in which borylation occurs *cis* to the smaller methyl group, consistent with low diastereoselectivity. Next, they explored fused bicyclic cyclopropanes which underwent borylation in moderate to high yields, preferentially activating the tertiary C─H bond at the ring fusion over the more sterically accessible secondary cyclopropyl C─H bond. This was rationalized with the increased *s*‐character of the former over the latter. Consistent with the electronic effect seen with the spiro substrates, the *N*‐triflyl‐fused cyclopropane exclusively formed the bridgehead boronic ester (**21.4b**) in quantitative yield. The same borylation strategy was also applicable to cyclobutanes, azetidines, and β‐lactams, again with high diastereoselectivity.

Complementary to this approach, Sawamura and coworkers in 2014 reported a heteroatom‐directed C─H borylation of cyclopropanes that proceeds with excellent regioselectivity and *cis*‐selective stereochemistry (Scheme 21c) [114]. The reaction is catalyzed by the silica‐supported monophosphane–Ir catalyst system Silica–SMAP. Cyclopropanes (**21.6a–c**) bearing functionalized pyridines, benzimidazoles, oximes, and representative benzoxazole, benzothiazole, *N*‐sulfonyl imine, and amide groups all proved suitable substrates. On the cyclopropane ring, methyl and gem dimethyl substitution, as well as a [4.1.0]heptane framework, were successfully borylated, the latter constituting the first reported catalytic borylation of a tertiary C–H bond. Interestingly, increasing the loading of the diboron reagent from 0.5 to 2.5 equivalence furnished a 1,2,3‐trisubstituted cyclopropane bearing two boron substituents, with all three substituents in a *cis* configuration. In every case, borylation occurred at the cyclopropane C─H bond located γ to the heteroarene or carbonyl N/O atom to give the *cis*‐configured borylcyclopropanes (**21.7a–c**).

In 2015, Itami and coworkers exploited a *cis*‐selective C─H borylation of cyclopropylamines as a key step toward the synthesis of 2‐arylcyclopropylamines, targets of interest in medicinal chemistry for developing LSD1 inhibitors (Scheme 21d) [115]. The Hartwig borylation conditions were unsuccessful for unprotected cyclopropylamines, so the authors employed *N*‐cyclopropylpivalamides (**21.8**), using the pivaloylamide group as a directing group for C─H activation. After optimizing the borylation, they combined it with a subsequent Suzuki–Miyaura coupling that efficiently transformed the resulting borylcyclopropanes (**21.9**) into a range of 2‐arylcyclopropylamines.

Complementing these iridium‐based approaches to C─H borylation of cyclopropanes, Yu and coworkers in 2016 disclosed a palladium catalyzed borylation of primary β C(sp^3^)─H bonds in carboxylic acid derivatives and secondary C(sp^3^)─H bonds in a range of carbocyclic rings, including cyclopropanes (Scheme 21e) [116]. The reaction employed Pd(OAc)_2_ ligated by quinoline (**L6**), successfully borylating cyclopropylamides (**21.10**) with a preference for *cis* diastereomer borylcyclopropanes (**21.11**). An enantioselective variant was developed by the same group in 2017 through the use of chiral acetyl‐protected aminomethyloxazoline ligands, though application to cyclopropanecarboxylic amides afforded the borylated product in low yield despite excellent ee [117].

Addressing the yield limitations of the 2017 report, Xu and coworkers reported an amide directed, enantioselective Ir catalyzed C(sp^3^)─H borylation of cyclopropanes (**21.12**) using modified chiral bidentate boryl ligands (CBLs) in 2019 (Scheme 21f) [118]. Increasing the steric bulk on the aryl substituent of the CBL (**L7**) and introducing a *tert*‐butyl group on the other aryl ring delivered the borylated products (**21.13**) in excellent yield and ee. The amide scope included diethylamide, pyrrolidine, piperidine, and piperazine substituents. Variation of the α substituent showed broad tolerance for *meta*‐ and *para*‐ substituted aryl groups, as well as several α alkyl groups. Cinacalcet and estradiol derived cyclopropane carboxamides also underwent efficient borylation, albeit at elevated temperature (80°C). In 2022, building on Itami's 2015 study, Xu et al. reported an imide directed, enantioselective C(sp^3^)─H borylation of aminocyclopropanes (**21.14**) using CBL(**L8**)/Ir catalysis (Scheme 14g) [119]. Various nitrogen protecting groups were examined, with succinimide giving the highest yield but initially low ee, a performance attributed to its conformational rigidity and favorable six membered cyclometallation geometry. Subsequent optimization of the CBL ligand increased the ee of borylcyclopropanes (**21.15**) from 2% to 94%. The reaction tolerated a range of cyclic imide directing groups and both α aryl and α alkyl substituents, extending alkyl scope beyond the 2019 work to include longer linear chains, bulky *tert*‐butyl groups, and diverse carbocycles. In 2023, the same group extended this strategy to an ether‐directed, enantioselective C–H borylation of cyclopropanes using CBL/Ir catalysis [120].

As an alternative to these transition‐metal‐mediated protocols, Kananovich and co‐workers in 2019 reported a site‐ and stereoselective lithiation of tertiary cyclopropylamines **21.16a–b** to access multifunctionalized cyclopropanes, including borylcyclopropanes **21.17a–b** (Scheme 21h) [121]. To favor β‐lithiation over α‐lithiation or directed *ortho*‐metalation, various N‐protecting groups were evaluated via deuterium quenching experiments; pivaloylamides proved optimal for regioselective β‐lithiation. Computational studies underscored the directing group's conformational orientation as key to β‐selectivity, with tetramethylsuccinimides emerging as a second viable protecting group. A range of electrophiles was then employed, enabling diverse cyclopropane C–H functionalization with carbon, silicon, boron, iodine, and sulfur groups [121].

## 5 Hydroboration Approaches to Borylcyclopropanes

Hydroboration represents one of the most atom‐economical and operationally straightforward routes to organoboron compounds, and its application to strained unsaturated cyclopropane precursors offers a direct and selective entry into borylcyclopropanes. Unlike the carbene‐based and nucleophilic cyclization strategies discussed previously, hydroboration routes exploit the inherent reactivity of the carbon–carbon double bond within cyclopropene and alkylidenecyclopropane frameworks, delivering the boryl group with predictable regio‐ and stereoselectivity governed by the strained ring geometry.

### 5.1 Hydroboration of Cyclopropenes

**SCHEME 22:**
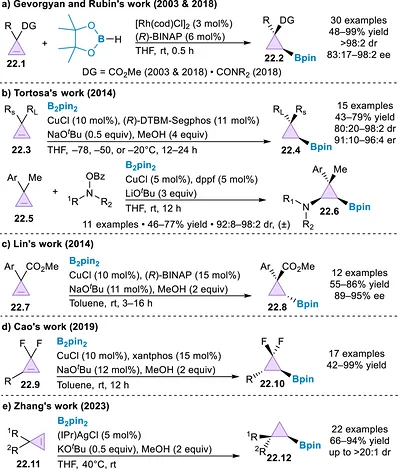
Hydroboration of cyclopropenes.

Gevorgyan, Rubin and coworkers reported the first asymmetric hydroboration of cyclopropenes to access chiral borylcyclopropanes (**22.2**) in 2003 (Scheme 22a) [122]. This rhodium catalyzed process afforded products in nearly quantitative yields with excellent diastereo‐ and enantioselectivity. The [Rh(COD)Cl]_2_/(R) BINAP system reacted efficiently with disubstituted cyclopropenes (**22.1**) bearing ester substituents, while substrates containing methoxymethyl groups required the (*R,R*)‐Et‐BPE ligand to achieve high enantioinduction. In contrast, trisubstituted cyclopropenes were not tolerated under these conditions. Following this initial advance, Rubin and coworkers expanded the scope in 2018 to include cyclopropenes bearing either ester or amide directing groups [123]. The introduction of Lewis basic amide functionality enabled stronger chelation to the rhodium center, consistently delivering high diastereo‐ and enantioselectivity across a broader range of substrates.

In 2014, Tortosa and coworkers disclosed a copper(I) catalyzed synthesis of enantioenriched borylcyclopropanes (**22.4** and **22.6**) based on a chiral Cu–boryl complex operating under directing group free conditions (Scheme 22b) [124]. Dimerization of the intermediate initially eroded yield, but could be suppressed effectively by addition of the cyclopropene and MeOH to the preformed Cu–boryl complex at −78°C and allowing the hydroboration to proceed at between −78°C to −20°C depending on the substrate. The borylcyclopropanes were formed in excellent yield and diastereoselectivity. Use of (*R*)‐DTBMSegphos delivered the best combination of yield, diastereomeric ratio, and enantiomeric ratio. The protocol tolerated a broad range of 3,3‐disubstituted cyclopropenes (**22.3** and **22.5**) bearing aryl, heteroaryl, alkyl, methoxymethyl, and spirocyclic substituents. Expanding beyond proton trapping, interception of the organocopper intermediate with *O*‐benzoyl‐*N,N*‐dialkylhydroxylamines at room temperature provided cyclopropylaminoboronic esters without detectable dimerization, and was applicable to piperidine, pyrrolidine, tetrahydroisoquinoline, morpholine, and piperazine derivatives. Marek and co‐workers were able to adapt these conditions for the copper‐catalyzed hydro‐ and carboborylation of substituted cyclopropenyl esters in their borylcyclopropanation methodology that they applied toward stereospecific ring‐opening [125].

Published 1 month earlier, Lin and coworkers described a related copper catalyzed asymmetric hydroboration of aryl and methyl ester disubstituted cyclopropenes **22.7** (Scheme 22c) [126]. Although the substrate scope was somewhat limited, the reaction proceeded smoothly at room temperature to afford optically active *trans*‐borylcyclopropanes (**22.8**) in moderate to high yields and with excellent enantioselectivity.

A similar method was described by Cao and coworkers in 2019, involving efficient copper catalyzed hydroboration of difluorocyclopropenes (**22.9**) for the synthesis of *trans*‐difluorocyclopropyl boronic esters (**22.10**), only the second publication describing access to these compounds (Scheme 22d) [127]. Reaction performance was strongly solvent dependent, with toluene providing the highest yields. The method exhibited broad functional group tolerance, accommodating both electron withdrawing and electron donating aryl substituents, as well as thiophene, naphthalene, and alkyl derivatives.

Departing from copper‐mediated approaches, Zhang and coworkers reported a silver(I)‐catalyzed diastereoselective hydroboration of cyclopropenes (**22.11**) using commercially available NHC‐AgCl catalysts in 2023 (Scheme 22e) [128]. (IPr)AgCl was identified as the optimal catalyst, delivering cyclopropylboronates in up to 94% yield and >20:1 dr under mild conditions tolerant of open air. The reaction accommodated a broad range of 3,3‐disubstituted cyclopropenes bearing aryl, heteroaryl, and aliphatic substituents, with electron‐deficient cyclopropenes shown by competition experiments to react preferentially over electron‐rich analogs. Mechanistic studies, including a control experiment with TEMPO argued against a radical pathway. Rather, a kinetic isotope study supported a pathway involving formation of an NHC–Ag–Bpin species, addition across the cyclopropene double bond to generate a boryl organosilver intermediate, and turnover‐limiting protonation by methanol to deliver the product (**22.12**).

### 5.2 Hydroboration of Alkylidenecyclopropanes

**SCHEME 23:**
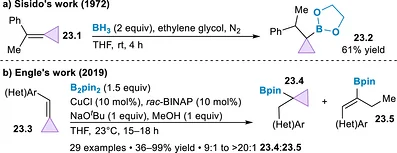
Hydroboration of alkylidenecyclopropanes.

In 1972, Sisido and coworkers disclosed the first synthesis of borylcyclopropanes through the hydroboration of alkylidene/benzylidenecyclopropanes (Scheme 23a). Treatment of benzylidenecyclopropane **23.1** with borane under a nitrogen atmosphere, followed by quenching with excess ethylene glycol, furnished borylcyclopropane **23.2**, providing the initial precedent for boron incorporation into the alkylidenecyclopropane framework [129].

Building on Sisido's foundational 1972 precedent, Engle and coworkers demonstrated in 2019 that borylcyclopropanes could be synthesized via copper catalyzed hydroboration of benzylidenecyclopropanes (Scheme 23b) [130]. Competition between borylcyclopropanes and alkenylboronic esters arose from divergent protodecupration and β elimination pathways, respectively. Phosphine ligands with larger bite angles, such as (*rac*)‐BINAP, favored formation of borylcyclopropanes over alkenylboronic esters. The reaction tolerated a range of benzylidenecyclopropanes (**23.3**), including more complex substrates bearing heterocycles common in biologically active molecules, and generally delivered products in excellent yields with good chemoselectivity toward borylcyclopropane (**23.4**) formation.

## 6 Miscellaneous Borylcyclopropanation Methods

The synthetic routes to borylcyclopropanes discussed thus far share a common reliance on well‐defined mechanistic paradigms such as carbene transfer, nucleophilic cyclization, C─H activation, and hydroboration. This section collects a set of complementary approaches that, while more difficult to classify, provide valuable and often unique access to the borylcyclopropane framework, ranging from functionalization of preformed cyclopropane scaffolds and strain‐release strategies to boron‐centered rearrangements and addition reactions.

### 6.1 Cyclopropane Functionalization via Leaving Group Substitution

**SCHEME 24:**
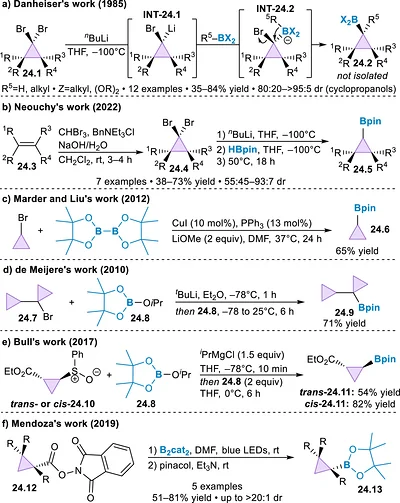
Cyclopropane functionalization via leaving group substitution.

In the first report on the synthesis of cyclopropylboranes via metal–halogen exchange in 1985, Danheiser and Savoca described the conversion of dibromocyclopropanes into secondary and tertiary cyclopropylboranes (Scheme 24a) [131]. The sequence began with a stereospecific suprafacial addition of dibromocarbene to an olefin to furnish dibromocyclopropane derivative **24.1**, which was subjected to metal–halogen exchange with *^n^* butyllithium to give a *gem*‐lithiobromocyclopropane intermediate (**INT‐24.1**). Addition of this intermediate to various borane reagents (such as catecholborane, trimethylborane, triethylborane, etc.) formed a boronate complex (**INT‐24.2**) that underwent a 1,2‐metalate shift (Matteson–Pasto rearrangement) to generate the desired cyclopropylborane **24.2**. In situ oxidation with alkaline hydrogen peroxide provided an efficient route to secondary and tertiary cyclopropanols with high degrees of stereoselectivity. The stereochemical outcome reflects a sequence of well‐defined steps: in the *gem‐*lithiobromocyclopropane (**INT‐24.1**), the lithium resides either *syn* to a chelating substituent or on the more sterically encumbered face of the cyclopropane ring; electrophilic substitution by the borane occurs with retention at the carbon–lithium bond, the subsequent 1,2‐migration proceeds with inversion at the migrating cyclopropyl carbon, and the final oxidation step proceeds with full retention of configuration.

Elaborating on the Matteson–Pasto rearrangement, Neouchy et al. in 2022 set out to isolate the borylcyclopropane intermediate from Danheiser's work by omitting the oxidative step and employing pinacolborane in place of catecholborane or trialkylboranes (Scheme 24b) [132]. Dibromocyclopropanation of alkenes **24.3** was performed using bromoform as the carbene precursor, followed by a Matteson–Pasto rearrangement sequence with *^n^* butyllithium and pinacolborane. In this way, dibromocyclopropanes (**24.4**) were transformed directly into synthetically versatile cyclopropyl pinacol boronic esters (**24.5**), rather than into cyclopropanols as in Danheiser's original protocol, though with somewhat diminished diastereoselectivity.

Steering away from dibromocyclopropanes and toward alkyl halides more broadly, Steel, Marder, and Liu groups in 2012 pioneered an unprecedented copper‐catalyzed borylation of unactivated alkyl halides and pseudohalides with diboron reagents to prepare primary and secondary alkylboronic esters (Scheme 24c) [133]. Among the wide variety of primary and secondary alkyl electrophiles tested, cyclic secondary bromides such as bromocyclopropane were also smoothly converted to cyclopropylboronic acid pinacol ester **24.6** in 65% yield.

In an alternative metal–halogen exchange approach, de Meijere et al. reported a bromine–lithium exchange reaction of 1‐bromo‐1‐cyclopropylcyclopropane (**24.7**) with *tert*‐butyllithium, generating 1‐lithio‐1‐cyclopropylcyclopropane that could be trapped with a range of electrophiles to furnish 1‐substituted bicyclopropyl derivatives (Scheme 24d) [134]. Among these, the corresponding (1‐cyclopropylcyclopropyl)boronic ester **24.9** was obtained with 71% yield.

Using a similar metalation strategy, Bull and coworkers developed a sulfoxide–magnesium exchange followed by electrophilic trapping in 2017 as part of a larger study to access diverse cyclopropane containing lead‐like compounds and fragments (Scheme 24e). This method provided a convenient and efficient platform for cyclopropane functionalization [135]. In this work, the (*cis*)‐ and (*trans*)‐sulfinyl esters (**24.11**) were synthesized as racemates with an inconsequential mixture of sulfoxide stereoisomers. They were converted, via sulfoxide–magnesium exchange, to the corresponding cyclopropyl Grignard reagent, which was then trapped with isopropoxy dioxaborolane (**24.8**) to furnish the corresponding cyclopropyl boronic esters (*trans* **‐** and *cis*‐**24.11**). In 2021, Huang, Marder and coworkers reported a base‐mediated radical borylation of alkyl sulfones using B_2_neop_2_ as the boron source under transition metal‐free conditions, including a cyclopropyl sulfone substrate in the scope that provided direct access to cyclopropylboronic ester [136].

Diverging from direct borylation strategies, Mendoza and coworkers disclosed in 2019 an enantioselective cyclopropanation of unfunctionalized aliphatic alkenes using a diazo compound containing an *N*‐hydroxyphthalimide (NHPI) ester, exploiting the dual capacity of NHPI esters to act as acyl electrophiles and radical precursors (Scheme 24f) [137, 138]. The resulting enantioenriched redox‐active cyclopropane carboxylates (**24.12**) could then be diversified via decarboxylative transformations to borylcyclopropanes (**24.13**), selenocyclopropanes, cyclopropylamines, cyclopropanols, and other densely functionalized derivatives.

### 6.2 Ring‐Opening of Bicyclo[1.1.0]Butane Derivatives

**SCHEME 25:**
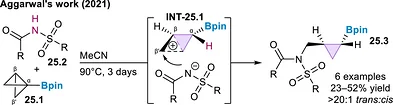
Borylcyclopropanes from ring‐opening of bicyclo[1.1.0]butane derivatives.

Investigating the reaction of bicyclo[1.1.0]butyl pinacol boronic ester (**25.1**) with nucleophiles, Noble, Aggarwal and Guo in 2021 demonstrated that both borylated cyclobutanes and cyclopropanes could be accessed, depending on the nucleophile (Scheme 25) [139]. While various alcohols, carboxylic acids, thiols and sulfonamides yielded α‐heteroatom‐substituted cyclobutyl boronic esters, sterically hindered bis‐sulfonamides and *N*‐tosylbenzamides (**25.2**) reacted with **25.1** and at the β’‐position to give 1,2‐disubstituted cyclopropyl boronic esters (**25.3**) in moderate yields and with complete *trans*‐selectivity. The proposed cyclopropanation mechanism involves protonation of **25.1** at the α‐carbon to generate the nonclassical cyclopropylcarbinyl cation **INT‐25.1**, followed by reaction with the aza‐anion to give **25.3**. The authors attributed the divergence in reactivity to the reduced nucleophilicity and increased steric bulk of bis‐sulfonamides and *N‐*tosylbenzamides, which disfavor boronate formation and 1,2‐migration to a cyclobutane, instead channeling the reaction through the cyclopropanation pathway.

### 6.3 Alkylboration of Cyclopropenes/Benzylidenecyclopropanes

**SCHEME 26:**
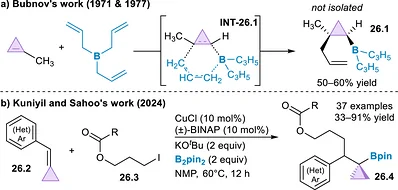
Borylcyclopropanation via alkylboration of cyclopropenes and benzylidenecyclopropanes.

In a pioneering 1971 contribution, Bubnov, Kazansky and coworkers demonstrated that 1‐methylcyclopropene reacted with triallylborane to generate diallyl(2‐allyl‐2‐methylcyclopropyl)borane **26.1** (Scheme 26a) [140]. The dominant pathway involved *cis*‐addition of the allyl and diallylboryl moieties across the cyclopropene double bond, accompanied by an allylic rearrangement via **INT‐26.1**. In 1977, the same group extended the chemistry to 1,3,3‐trimethylcyclopropene and triallylborane to access the corresponding borylcyclopropane [141]. Although the borylcyclopropane intermediates (e.g., **26.1**) were not isolated in either case, their formation was inferred from methanol‐promoted cleavage of the allyl–boron bonds followed by oxidation to the corresponding cyclopropanols, as well as from IR and ^1^H NMR spectroscopic data.

In another 1,2‐alkylboration approach, Kuniyil, Sahoo and coworkers in 2024 probed accessing diverse tertiary cyclopropyl boronic esters via benzylidenecyclopropanes under copper catalysis (Scheme 26b) [142]. Benzylidenecyclopropane (**26.2**) coupled with β─H‐containing alkyl iodides (**26.3**) and B_2_pin_2_ to afford C(sp^3^)‐enriched tertiary cyclopropyl boronic esters (**26.4**) in modest‐to‐high yields. The protocol exhibited broad substrate tolerance, encompassing a wide array of aryl and heteroaryl benzylidenecyclopropanes together with structurally diverse alkyl iodides, including simple and homobenzylic variants, those bearing aliphatic or (un)substituted aromatic benzoate esters, phthalimide or carbazole units, and derivatives of drugs and natural products. Mechanistic investigation using radical clock experiments, Hammett analysis, and DFT studies supported a polar manifold rather than a radical pathway, featuring boryl cupration followed by rate‐limiting S_N_2 type C─C bond formation.

### 6.4 1,2‐Metalate Rearrangement of Cyclopropenylboronates

**SCHEME 27:**
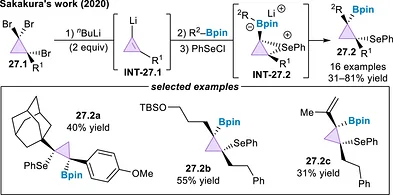
Borylcyclopropanes via 1,2‐metalate rearrangement of cyclopropenylboronates.

In 2020, Mizoguchi, Seriu, and Sakakura reported the conversion of tribromocyclopropanes into densely functionalized β‐selenocyclopropylboronic esters via the 1,2‐metalate rearrangement of a boronate complex (Scheme 27) [143]. The sequence proceeds in three steps: (1) treatment of tribromocyclopropanes (**27.1**) with *^n^* butyllithium to generate a cyclopropenyllithium species (**INT‐27.1**), (2) in situ trapping of this intermediate with an organoboronic ester to form a cyclopropenylboronate ate complex, and (3) activation of the cyclopropene C═C bond with phenylselenenyl chloride, which triggers a stereospecific 1,2‐metalate rearrangement to afford the β‐selenocyclopropylboronic ester **27.2** via **INT‐27.2**. DFT calculations support the intermediacy of a seleniranium ion intermediate **INT‐27.2**. The method tolerates a variety of aryl and alkyl boronic esters, and the tribromocyclopropane component can bear diverse R groups, including adamantyl (**27.2a**), phenyl, silyl ethers (**27.2b**), an alkenyl moiety (**27.2c**), and a tribromocyclopropane fused to a cycloheptane ring.

## 7 Applications of Borylcyclopropanes

Having surveyed the landscape of synthetic routes to borylcyclopropanes, this section turns to their downstream utility. The versatile C─B bond serves as a springboard for diverse applications, starting with their direct incorporation into medicinal chemistry programs and natural product synthesis. They also serve as precursors in ring‐opening and ring‐expansion reactions that deliver enantioenriched acyclic organoboron structures, cyclopentenes, cycloheptadienes, and alkylidenecyclopropanes, underscoring the value of the borylcyclopropane motif as a versatile synthetic intermediate in both complex molecule synthesis and structural diversification.

### 7.1 Borylcyclopropanes in Medicinal Chemistry

**SCHEME 28:**
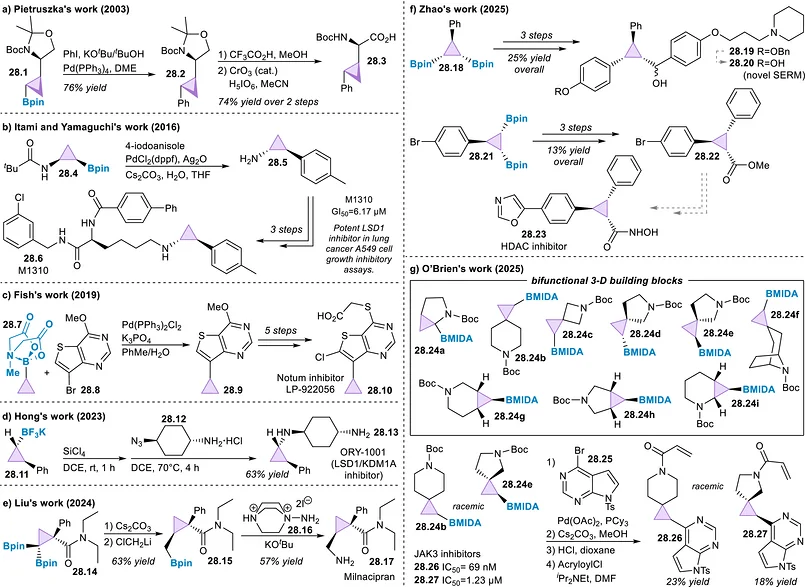
Borylcyclopropanes in medicinal chemistry.

Cyclopropane amino acids are noncanonical residues increasingly valued in medicinal chemistry as building blocks for therapeutic and peptidomimetic scaffolds [6]. Their rigid geometry imparts conformational constraint that enables highly efficient and selective receptor interactions. The effect has been exemplified by cyclopropane amino acid motifs in conformationally constrained peptide analogues, in bioactive natural products like coronatine, and in antiviral APIs such as Grazoprevir and Simeprevir [3, 144, 145]. Pietruszka et al. showcased in 2003 how cyclopropylboronic esters serve as versatile precursors to unusual amino alcohols and amino acids, leveraging Garner aldehyde as a chiral control element (Scheme 28a) [54]. In their three‑step sequence, both diastereomers of Garner aldehyde‑derived borylcyclopropanes (**28.1**) underwent Suzuki–Miyaura coupling with aryl partners to form **28.2**, followed by N‑deprotection to amino alcohols and subsequent oxidation to the corresponding cyclopropane amino acids (**28.3**). This approach provides stereodefined, conformationally rigid building blocks primed for incorporation into medicinally relevant peptide frameworks.

LSD1 inhibitors serve as powerful probes to dissect the biological roles of this epigenetic enzyme in cancer, globin disorders, and metabolic disease; they are also being advanced as potential therapeutics [146]. In 2016, Suzuki, Itami, and Yamaguchi leveraged C─H borylation of cyclopropylamine followed by cross‐coupling of the resulting borylcyclopropylamines to perform a rapid SAR campaign on arylcyclopropylamine LSD1 inhibitors, enabling late‐stage, modular installation of diverse aryl groups (Scheme 28b) [147]. Using this borylcyclopropane‐based platform, they accessed more than 45 analogues, including biaryl derivatives, and ultimately identified M1310 (**28.6**) as a potent inhibitor that induced G_0_/G_1_ arrest and apoptosis in A549 lung cancer cells and showed clear antitumor efficacy in an A549 xenograft model. M1310 was assembled from a borylcyclopropane precursor (**28.4**), which first underwent a Suzuki–Miyaura coupling to furnish the corresponding arylcyclopropylamine (**28.5**). As is typically observed, the Suzuki–Miyaura coupling occurred with retention of stereochemistry; however, under these conditions the cyclopropyl amide stereocenter underwent epimerization through an unknown mechanism [148]. A short sequence of functional group manipulations then delivered the final inhibitor. Notably, M1310 outperformed the reference inhibitor NCD38, further underscoring how the C─H borylation/Suzuki–Miyaura diversification of borylcyclopropanes can rapidly deliver clinically relevant leads.

LP‐922056 (**28.10**) is a clinically validated Notum inhibitor for Wnt pathway modulation and osteoporosis treatment, and its improved synthesis by Fish and coworkers in 2019 exemplifies the pharmaceutical utility of borylcyclopropanes in drug synthesis (Scheme 28c) [149]. The scalable route featuring a Suzuki–Miyaura cross‐coupling of a MIDA‐boronic ester cyclopropyl reagent **28.7** with bromide **28.8** to install cyclopropyl moiety on the thienopyrimidine core, yielding **28.9**. The MIDA protecting group provided superior stability and simplified chromatographic purification relative to the free boronic acid, enabling reliable multigram‐scale production of LP‐922056 (**28.10**) in good efficiency and high purity. Mouse pharmacokinetic studies confirmed its peripheral restriction (brain/plasma ratio = 0.01), ideal for selective Wnt modulation outside the CNS.

In 2020, Kleban et al. showcased the utility of cyclopropyl boronic derivatives in parallel library synthesis by preparing a 96‐member set of sp^3^─rich scaffolds via Suzuki–Miyaura coupling with (hetero)aryl halides [150]. They achieved a 68% success rate across the library with an average isolated yield of 30%. Physicochemical profiling of the products indicated general compliance with lead‐likeness criteria, with only a modest cLogP drift between the 140 planned and 96 successfully synthesized library members (2.98 vs. 3.00, respectively), underscoring the suitability of cyclopropyl‐containing motifs for medicinal chemistry applications. The following year, the same group reported a scalable 4–5 steps sequence for multigram synthesis of oxa‐ and aza‐bicyclo[*n*.1.0]alkan‐1‐yl trifluoroborates, motivated by the pharmaceutical relevance of the azabicyclo[n.1.0]alkane scaffold and its utility as a building block in C─C coupling reactions [151]. The method employed Pd(OAc)_2_‐catalyzed diazomethane cyclopropanation, although sulfur‐containing analogues were incompatible with this step. The final conversion of pinacol boronic esters to the target trifluoroborates was performed on up to ∼50 g scale in a single run, all while maintaining 10%–41% overall yield across the sequence, demonstrating the practical viability of these building blocks for medicinal chemistry efforts.

In 2022, Zhou and coworkers assembled a library of structurally diverse spirocyclopropylthiooxindoles through Rh‑catalyzed stereoselective cyclopropanation of diazothiooxindoles with α‑functionalized styrenes, followed by systematic evaluation of antiproliferative activity against HCT116, SJSA‑1, and MCF‑7 cell lines (see Section 2.1.3, Scheme 4c) [64]. Seventeen analogues exhibited IC_50_ values below 50 µM, with a CH_2_F‑substituted spirocyclopropylthiooxindoles emerging as the standout hit against SJSA‑1 osteosarcoma cells. Although the boron‑containing variants did not rank among the top performers, this work establishes a proof‑of‑concept for incorporating boryl pharmacophores into bioactive spirocyclopropane frameworks, expanding the accessible chemical space for future medicinal optimization.

In 2023, Hong and coworkers demonstrated the utility of their metal‐free borylcyclopropanation protocol through a concise synthesis of the LSD1 inhibitor ORY‐1001 (iadademstat), currently in clinical trials for acute myeloid leukemia and small cell lung cancer (Scheme 28d) [16]. The cyclopropyltrifluoroborate salt (**28.11**) was converted in situ to a dichloroborane, which underwent stereoretentive amination with azide (**28.12**) to deliver the arylcyclopropylamine as the HCl salt (**28.13**) in 63% yield over two steps.

Similarly, Liu and coworkers in 2024 demonstrated the utility of their stereoselective *gem*‐diborylcyclopropane synthesis through a direct, diastereoselective route to the antidepressant milnacipran (Scheme 28e) [21]. Borylcyclopropane (**28.14**) underwent stereoselective protodeborylation followed by Matteson homologation to afford intermediate (**28.15**), which was then converted to milnacipran (**28.17**) via amination with H_2_N‐DABCO salt (**28.16**) [152].

In 2025, Zhao and coworkers elaborated their diborylcyclopropanes (**28.18**, **28.21**) into key intermediates (**28.19**, **28.22**) for a novel SERM (selective estrogen receptor modulator) **28.20** and an HDAC (histone deacetylase) inhibitor **28.23**, respectively (Scheme 28f) [19]. These syntheses illustrate the versatility of diborylcyclopropanes as late‐stage diversification handles in medicinal chemistry campaigns targeting diverse therapeutic modalities.

In a 2025 landmark contribution to fragment‑based drug discovery (FBDD), Gómez‑Angel, O'Brien, and coworkers introduced nine sp^3^─rich, bicyclic cyclopropane‑based bifunctional building blocks (**28.24a–i**) that systematically explore distinct 3D vectors from 2D fragment hits (Scheme 28g) [153]. Each compound features a cyclopropyl MIDA boronic ester for reliable Suzuki–Miyaura fragment coupling (delivering crystalline, bench‑stable products) and a protected cyclic amine for *N*‑capping group installation. The cyclopropane‐containing bicyclic core locks the relative 3D orientation of the orthogonal handle pair, which systematically explores distinct vectors from 2D fragment hits and enabled a 32‐member lead‐like library. Proof‐of‐concept application delivered novel, selective JAK3 inhibitors **28.26** and **28.27** (IC_50_ = 69 nM and 1.23 µM, respectively) from a pyrrolopyrimidine fragment hit and scaffolds **28.24b** and **28.24e**, which also showed good metabolic stability. This work showcases how borylcyclopropane building blocks solve the longstanding 3D elaboration bottleneck in FBDD efforts. With all nine scaffolds now commercially available, this platform is a potentially enabling tool for hit‐to‐lead optimization for medicinal chemists.

### 7.2 Borylcyclopropanes in Natural Product Synthesis

**SCHEME 29:**
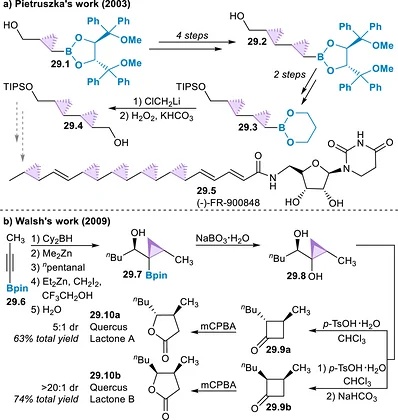
Borylcyclopropanes in natural product synthesis.

A report by the Pietruszka group in 2000 leveraged optically active, diol‐protected cyclopropylboronic esters en route to a known advanced intermediate **29.4** of the natural product jawsamycin (FR‐900848) **29.5**, a pentacyclopropane antifungal nucleoside from *Streptoverticillium fervens* (Scheme 29a) [3, 154]. Enantiomerically pure borylcyclopropane **29.1** was advanced through Wittig olefination, alcohol oxidation/reduction, and Simmons–Smith cyclopropanation (four steps total) to furnish alcohol **29.2**. Next, alcohol TIPS protection and transesterification to the dioxaborinane led to **29.3**, followed by a Matteson homologation and oxidation, which delivered the target intermediate **29.4**. This work established the exceptional stability of diol‐protected cyclopropylboronic esters under orthogonal manipulations, enabling their first use toward oligocyclopropane natural product synthesis. In 2021, Baran and coworkers accessed a different known intermediate of jawsamycin via cyclopropyl‐Bpin methyl ester, demonstrating an alternative retrosynthetic approach to the oligocyclopropane core [105].

In the 2009 Simmons–Smith borylcyclopropanation report, Walsh and coworkers extended their methodology to a diastereoselective synthesis of 2,3‐disubstituted cyclobutanones via pinacol‐type rearrangements of oxidized tetrasubstituted borylcyclopropanes (Scheme 29b) [71]. Catalytic *p*‐TsOH promoted rearrangement of the α‐hydroxycyclopropyl carbinols to give *trans*‐2,3‐disubstituted cyclobutanones, whereas BF_3_·OEt_2_ delivered the corresponding *cis* isomers, both in excellent diastereoselectivities and good yields. They showcased the synthetic utility of this manifold in a diastereoselective synthesis of (±)‐quercus lactones A and B. Thus, 1,1‐bimetallic‐mediated borylcyclopropanation of a methylalkynyldioxaborolane (**29.6**) furnished the α‐hydroxycyclopropyl boronic ester (**29.7**), which was oxidized to the corresponding α‐hydroxycyclopropyl carbinol (**29.8**). Subsequent acid‐promoted pinacol rearrangements provided the *cis*‐ and *trans*‐cyclobutanones (**29.9a** and **29.9b**, respectively), with the *trans* cyclobutane arising via rearrangement/isomerization following the initial pinacol rearrangement. These cyclobutanones then underwent Baeyer–Villiger oxidation to deliver quercus lactone A (**29.10a**) in 5:1 dr and quercus lactone B (**29.10b**) in >20:1 dr. Together, these studies firmly establish borylcyclopropanes as versatile intermediates for accessing densely functionalized, stereochemically rich small‐ring motifs in natural product synthesis.

### 7.3 Enantioenriched Acyclic Structures From Borylcyclopropanes

There is substantial demand for acyclic fragments bearing multiple stereocenters, though stereoselective construction remains challenging from conformational flexibility and distorted bond geometries. Fortunately, stereospecific C─C bond cleavage of functionalized cyclopropanes has emerged as a powerful and increasingly popular strategy to address this challenge in recent years [155].

**SCHEME 30:**
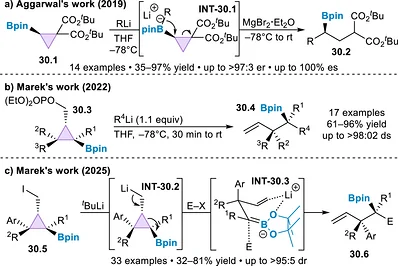
Enantioenriched cyclic structures from borylcyclopropanes.

The Aggarwal group found that cyclopropyl boronate complexes, unlike their bicyclo[1.1.0]butyl counterparts [156], failed to undergo 1,2‐metalate rearrangement/cyclopropyl ring‐opening of simple borylcyclopropanes under palladium‐mediated conditions (Scheme 30a) [157]. They proposed that the 1,2‐metalate rearrangement/ring‐opening sequence could be enabled by stabilizing the resulting carbanion through a diactivated borylcyclopropane (**30.1**) bearing a malonate substituent. This modification succeeded, although it required heating to 60°C for full conversion of boronate complex (**INT‐30.1**) to the optically active γ‐carbonyl boronic esters (**30.2**). The reaction tolerated diverse organolithium reagents, including alkyl, aryl, heteroaryl, and even steroidal migrating groups. It also enabled three‐component couplings with electrophiles such as methyl iodide, allyl iodide, or Eschenmoser's salt, which the malonate carbanion trapped instead of undergoing protonation.

Inspired by Aggarwal's work, Marek and coworkers in 2022 proposed that cyclopropyl boronic esters bearing a good leaving group, such as a phosphate, would readily undergo 1,2‐migration accompanied by carbon–carbon bond cleavage (Scheme 30b) [125]. Under these conditions, borylcyclopropanes (**30.3**) were converted in good yields and with complete or near‐complete stereospecificity into acyclic structures (**30.4**) featuring up to two vicinal stereocenters (tertiary or quaternary), using alkyl‐, aryl‐, and heteroaryl‐lithium reagents, while tolerating functional groups such as chlorides, silyl ethers, and aryl substituents. In a separate publication that same year, the group further demonstrated that the methodology is also compatible with alkynyl lithium nucleophiles [158].

Three years later, the same group employed a related borylcyclopropane scaffold (**30.5**, featuring an iodide substituent in place of phosphate) and devised a strategy initiating with lithium–iodine exchange to generate intermediate **INT‐30.2** (Scheme 30c) [22]. This lithiation promoted selective ring‐opening in the reverse direction via formal umpolung reactivity, delivering borata–alkene intermediate **INT‐30.3**, which was then trapped by electrophiles to afford acyclic boronic esters (**30.6**) with vicinal stereocenters. They identified optimal conditions for generating the α‐boryl carbanion **INT‐30.3** as addition of *^t^* BuLi at –95°C in diethyl ether favored lithium–halogen exchange over undesired ate‐complex formation. Cyclopropane ring‐opening and electrophilic trapping provided the homo‐allylic boronic esters **30.6**. Electrophilic trapping proved remarkably broad, encompassing primary alkyl iodides, allyl, methallyl, and benzyl electrophiles, acetone, sulfur‐based electrophiles, ClCH_2_Bpin, and suitably placed intramolecular traps. This methodology leveraged the anion‐stabilizing effect of boron and strain‐release‐driven fragmentation of readily accessible, stereodefined borylated cyclopropanes to deliver highly diastereoselective nucleophilic substitutions with exceptional chemo‐ and regioselectivity across a broad substrate scope and functional group tolerance.

### 7.4 Miscellaneous Applications of Borylcyclopropanes

In 1997, Charette et al. realized the first cyclopropyl–cyclopropyl cross‐coupling between a borylcyclopropane and cyclopropyl iodide [159]. The following year, Deng and coworkers reported the first detailed mechanistic studies on palladium‐catalyzed Suzuki–Miyaura cross‐couplings with chiral organoboron compounds, demonstrating stereoretentive transfer of the boron‐bound stereocenter's absolute configuration throughout the process [160]. In 2000, the same group explored the Suzuki‐type cross‐coupling reaction of cyclopropylboronic acids with acyl chlorides [161] and allylic bromides [162] to synthesize enantiomerically enriched cyclopropyl ketones (>90% ee) and stereodefined allyl‐substituted cyclopropanes, respectively. Pietruszka and coworkers provided the second example of a successful Pd‐catalyzed cyclopropane–cyclopropane cross‐coupling in 2004 using iodo‐boryl cyclopropanes. The approach provided the key advantage of retaining an intact boron moiety for subsequent elaboration of the resulting biscyclopropane [163, 164]. Since then, the Suzuki reactions of cyclopropylboronic MIDA esters [165], *gem*‐diborylcyclopropanes [166], and 1,2‐diborylcyclopropanes [167] have been reported.

**SCHEME 31:**
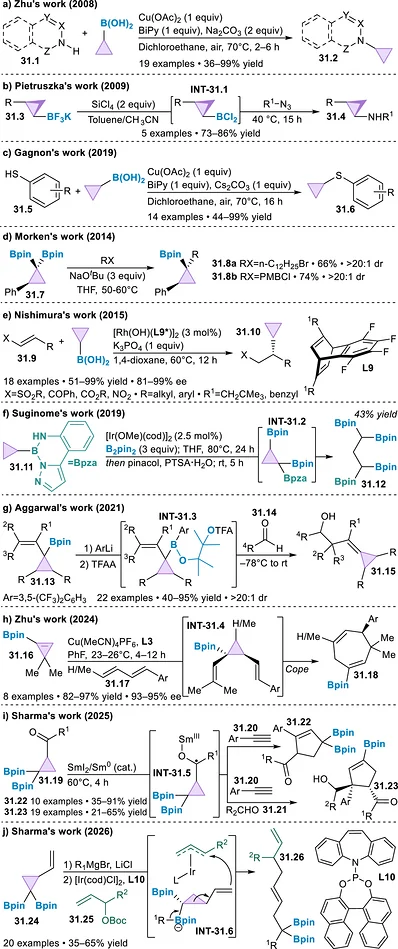
Miscellaneous applications of borylcyclopropanes.

The *N*‐cyclopropyl motif holds significant value in medicinal chemistry, yet traditional strategies, such as condensation of amines/anilines with cyclopropanone equivalents or functionalization of cyclopropylamines suffer from limited scope and low yields. Drawing inspiration from *N*‐cyclopropylation of cyclic amides and azoles via cyclopropylbismuth reagents [168], but wary of a failed attempt to couple cyclopropylboronic acids with amines under Chan‐Lam conditions [169], Tsuritani [170], and Zhu [171] groups independently discovered Cu(OAc)_2_‐catalyzed conditions enabling this transformation to access *N*‐cyclopropyl derivatives **31.2** (Scheme 31a). Tsuritani functionalized indoles and cyclic amides, while Zhu demonstrated broad applicability across azoles (e.g., imidazole, benzimidazole, pyrrole, carbazole, oxazolidinone, indole, and pyrazole), cyclic ureas, primary amides, and sulfonamides (**31.1**). In 2010, Zhu and coworkers expanded this methodology to the *N*‐cyclopropylation of anilines, primary and secondary aliphatic amines by cyclopropylboronic acid [172]. In 2009, the Pietruszka group introduced an efficient stereoretentive C─B to C─N transformation, converting enantiopure cyclopropyl trifluoroborates (**31.3**) to the corresponding cyclopropylamines (**31.4**) via a dichloride intermediate (**INT‐31.1**) and benzyl azide nucleophilic substitution (Scheme 31b) [173].

In 2019, Benoit, Fnaiche, and Gagnon reported a copper‐promoted *S*‐cyclopropylation of thiophenols (**31.5**) using cyclopropylboronic acid, delivering aryl cyclopropyl sulfides (**31.6**) in moderate to excellent yields under mild conditions with copper acetate catalysis (Scheme 31c) [174]. The protocol tolerates *ortho*‐, *meta*‐, and *para*‐substitution, as well as electron‐donating and ‐withdrawing groups on the thiophenol. It also accommodates potassium cyclopropyltrifluoroborate as an alternative boron source, providing a practical route to thioether motifs valuable in medicinal chemistry.

Turning to C─C bond formation through deborylation, Morken and coworkers disclosed a deborylative alkylation of *gem*‐diborylcyclopropanes (**31.7**) to furnish highly substituted tertiary boronic esters (**31.8a,b**) in 2014 (Scheme 31d) [12]. In this transformation, sodium *tert‐*butoxide was identified as a uniquely effective Lewis base, promoting smooth deborylation–alkylation even for internal geminal bis(boronic esters), which are typically less reactive. Applied to geminal diborylcyclopropanes, the method delivers alkylated products in good yields and with excellent stereocontrol (often >20:1 dr), albeit at elevated temperatures, thus providing a robust entry to densely substituted, stereodefined cyclopropane‐derived acyclic frameworks for downstream functionalization.

Extending C─B bond functionalization to conjugate addition, Takechi and Nishimura reported an enantioselective rhodium‐catalyzed 1,4‐addition of cyclopropylboronic acid to electron‐deficient olefins (**31.9**) using chiral diene ligands (**L9**) based on a tetrafluorobenzobarrelene framework, affording β‐cyclopropyl carbonyl, sulfonyl and nitro compounds (**31.10**) in high yields (up to 99%) and enantioselectivities (up to 99% ee) (Scheme 31e) [175]. This protocol exhibits excellent functional group tolerance including halides, heterocycles, and silyl ethers. The method offers a direct, stereo‐controlled route to cyclopropyl‐substituted acyclic motifs essential in medicinal chemistry and natural product synthesis.

In a complementary borylation strategy, Yamamoto, Ishibashi, and Suginome introduced a boryl‐directed Ir‐catalyzed C(sp^3^)─H borylation of alkylboronic acids using pyrazolylaniline as a temporary directing group on boron (Bpza), enabling site‐selective α‐, β‐, or γ‐borylation to access polyborylalkanes up to pentaboronated species (Scheme 31f) [176]. Notably, cyclopropyl‐Bpza (**31.11**) was converted to polyborylalkane **31.12** via intermediate **INT‐31.2**. The synthetic utility of these polyborylated molecules is underscored by their successful elaboration through cross‐coupling reactions [177] and Boron–Wittig olefination [178].

Hari, Aggarwal and coworkers introduced highly diastereoselective strain‐increasing allylboration reactions derived from vinyl cyclopropylboronic esters (**31.13**), enabling rapid construction of alkylidenecyclopropanes (**31.15**) from aldehydes or ketones (**31.14**) (Scheme 31g) [179]. To overcome unfavorable ring strain, they activated the boronic ester to a more reactive borinic ester intermediate (**INT‐31.3**), delivering the allylboration products in high yield and diastereoselectivity (>20:1 dr). This strategy forged exocyclic alkenes with quaternary centers while amplifying ring strain, highlighting borylcyclopropanes as versatile, stereodefined reagents for downstream ring‐opening cascades and complex acyclic array assembly.

Zhu and coworkers presented a potential route to cycloheptane cores found in various natural products via their enantioselective α‐boryl carbene‐transfer (see Section 2.2.4, Scheme 10c) [44]. Using aryl‐substituted conjugated dienes (**31.17**), the reaction with borylcyclopropene **31.16** proceeded through a tandem cyclopropanation/Cope rearrangement via a borylated divinylcyclopropane intermediate (**INT‐31.4**) to make cycloheptadienyl boronic esters **31.18** (Scheme 31h). The significance and synthetic utility of this divinylcyclopropane rearrangement as a route to cycloheptane frameworks in natural products was exemplified in Gaich and coworkers’ recent seminal total synthesis of (−)‐spiroaspertrione A, which employed the strategy to construct the spirobicyclo[3.2.2]nonane core [180].

In 2025, Sharma and coworkers merged ketyl radical chemistry with allylboration through strain‐release activation of diboryl cyclopropyl ketones **31.19** (Scheme 31i) [181]. In a one‐pot multicomponent reaction with alkynes **31.20** and aldehydes **31.21** (following SET and **INT‐31.5** formation) ketyl radicals underwent [2σ+2π] cycloaddition and selective ring opening, followed by stereo‐controlled allylboration to furnish densely functionalized cyclopentenes bearing adjacent quaternary/tertiary/secondary stereocenters (**31.22** or **31.23**) with high diastereoselectivity, including rare ketone‐functionalized γ,γ‐disubstituted allylic diborons (**31.22**). These multifunctional organoborons highlight the power of strain‐release diborylcyclopropanes for forging sterically congested scaffolds in a single operation.

Expanding on this strain‐release manifold, the same group introduced the strain‐release‐driven 1,2‐metalate rearrangement of vinylcyclopropyl diborons (**31.24**) in 2026 (Scheme 31j) [182]. Nucleophilic ring‐opening of the strained vinylcyclopropyl diborons triggered boronate migration, enabling multicomponent proximal/remote 1,5‐difunctionalization to generate densely functionalized homoallylic diboryl alkanes (allylic halides or branched 1,5‐dienes **31.26**) with high *E*‐selectivity. This process harnessed Ir‐catalyzed allylation (phosphoramidite ligand **L10**) of allylic carbonates (**231.25**) through intermediate **INT‐31.6**. The reaction proceeded efficiently with optically active ligands, yielding densely functionalized homoallylic diboryl alkanes (**31.26**). Enantioinduction could not be measured due to an inability to separate the product enantiomers chromatographically, and the protocol exhibited polarity inversion versus classical vinylcyclopropane reactions. A broad scope and enantioenrichment potential with chiral ligands position vinylcyclopropyl diborons as versatile linchpins for modular synthesis of bioactive scaffolds [183].

## 8 Summary and Outlook

This review has presented the first comprehensive survey of borylcyclopropane chemistry, systematically cataloging every synthetic route reported to date and documenting the scope of their downstream applications. The field has grown substantially over the past decade, driven by the recognition that the C─B bond and the strained cyclopropane ring together constitute a uniquely enabling combination that unlocks both distinctive cyclopropanation strategies and a broad spectrum of selective downstream transformations.

**FIGURE 1:**
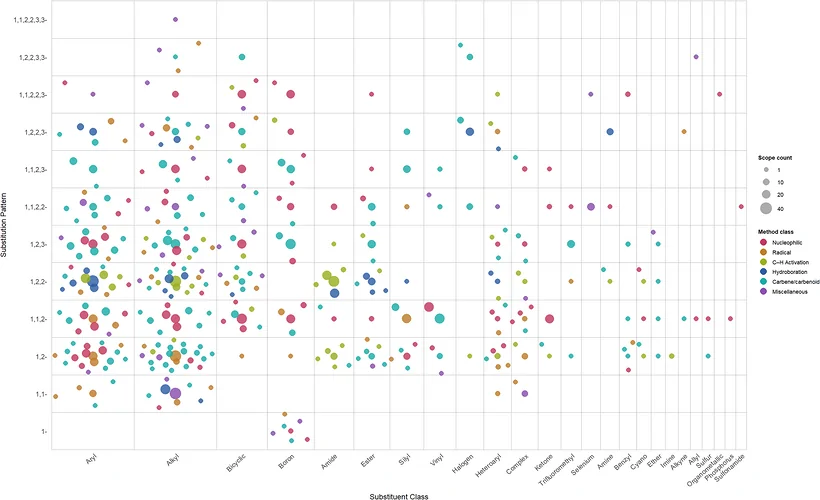
Landscape of borylcyclopropane synthesis mapped by substitution pattern, substituent class, and synthetic method. Matrix dot plot in which each dot represents one publication reporting at least one example with the corresponding substitution pattern (rows) and substituent class (columns). The boronic ester is considered part of the core scaffold and is not counted as a variable substituent, except when a second boron is present, in which case it is counted once. Dots are colored by synthetic method class: carbene/carbenoid (teal), nucleophilic cyclization (pink), radical/photocatalytic (orange), C─H activation (green), hydroboration (blue), and miscellaneous (purple). A publication contributes at most one dot per cell, although publications can be represented in multiple cells to capture the full substrate scope. Full data, including per‐paper assignments of substitution pattern, substituent class, and method class, are provided in Supporting Information.

To map the current boundaries of borylcyclopropane synthesis, Figure 1 surveys the substitution patterns and substituent classes accessible by existing methodology, providing an unprecedented overview of where the field stands (for details, see supporting information). The data reveal a field that has built strong foundations in simpler substitution patterns and common substituent classes, but where coverage drops sharply in both breadth and depth as synthetic complexity increases.

The 1,2‐ substitution pattern dominates the current literature with 47 publications and 86 unique publication–pattern–substituent combinations (each represented by one dot in Figure 1). Their prevalence reflects decades of carbene‐based cyclopropanation of vinylboronic esters dating back to Carboni's 1989 report, though these methods tend toward fewer examples per reported combination than more recent approaches. By contrast, the 1,1,2‐ and 1,2,3‐patterns each achieve comparable coverage of 58 and 49 publication–pattern–substituent combinations respectively from only 19 and 18 unique publications each, a concentration that reflects the breadth of individual contributions from nucleophilic *gem*‐diborylalkane cyclization, radical photocatalytic, and carbene‐based approaches that have collectively defined these patterns in recent years. C─H activation is concentrated in the 1,2,2‐ pattern, where the geometry of directed metalation naturally delivers borylation β to the directing group, while hydroboration is largely confined to the 1,2,2‐ and 1,1‐ patterns by its requirement for a C═C bond within or exocyclic to the cyclopropane ring. Literature coverage drops precipitously beyond tetrasubstituted patterns: the 1,1,2,2‐ pattern is documented by only 11 unique publications, the 1,1,2,2,3‐ and 1,2,2,3,3‐ by 5 each, and the hexasubstituted 1,1,2,2,3,3‐ by a single paper, reflecting the substantial synthetic challenge these targets present and highlighting that enantioselective catalytic routes to penta‐ and hexasubstituted borylcyclopropanes remain to be developed.

Aryl‐substituted borylcyclopropanes account for 85 unique publication–pattern–substituent entries encompassing 681 total substrate examples (over 8 per combination), whereas heteroaryl coverage amounts to only 23 such combinations encompassing 47 examples (approximately 2 per combination). This disparity reflects a tendency to develop and validate methodology around aryl substituents, with heteroaryl and medicinally relevant substituents yet to receive the same methodological attention. Other substituent classes with direct relevance to drug discovery such as amines, sulfonamides, and phosphonates are documented in only 4, 1, and 1 publication respectively, underscoring how much synthetic territory remains open.

The pace of development has accelerated markedly since 2019, and the convergence of nucleophilic *gem*‐diborylalkane chemistry, photocatalytic radical methods, enzymatic carbene transfer, and undirected C─H borylation has substantially diversified the diazo/metal‐carbene paradigm that defined the field's first 3 decades. Chief among the remaining challenges is the development of enantioselective routes to highly substituted targets, systematic expansion of heteroaryl and medicinally relevant substituent scope, and deeper exploitation of the C─B bond in stereospecific ring‐opening, strain‐release, and late‐stage diversification. While borylcyclopropanes have found increasing application in medicinal chemistry, their use in natural product synthesis remains strikingly limited relative to the structural complexity they can offer. As these challenges are addressed, borylcyclopropanes are poised to become indispensable building blocks in the synthesis of complex, stereodefined molecular architectures. It is our hope that this review serves not only as a comprehensive reference but as an inspiration for the next generation of borylcyclopropane chemistry, helping synthetic chemists identify reliable routes to their targets while also highlighting the open challenges that warrant the most attention (Supporting Information).

## Conflicts of Interest

The authors declare no conflicts of interest.

## Supporting information




**Supporting File**: anie73694‐sup‐0001‐Data.zip.

## Data Availability

The data that supports the findings of this study are available in the supplementary material of this article.
